# Hemotin, a Regulator of Phagocytosis Encoded by a Small ORF and Conserved across Metazoans

**DOI:** 10.1371/journal.pbio.1002395

**Published:** 2016-03-25

**Authors:** José I. Pueyo, Emile G. Magny, Christopher J. Sampson, Unum Amin, Iwan R. Evans, Sarah A. Bishop, Juan P. Couso

**Affiliations:** 1 Brighton and Sussex Medical School, University of Sussex, Brighton, United Kingdom; 2 School of Life Sciences, University of Sussex, Brighton, United Kingdom; 3 Department of Infection and Immunity and the Bateson Centre, University of Sheffield, Sheffield, South Yorkshire, United Kingdom; UT Southwestern Medical Center, UNITED STATES

## Abstract

Translation of hundreds of small ORFs (smORFs) of less than 100 amino acids has recently been revealed in vertebrates and *Drosophila*. Some of these peptides have essential and conserved cellular functions. In *Drosophila*, we have predicted a particular smORF class encoding ~80 aa hydrophobic peptides, which may function in membranes and cell organelles. Here, we characterise *hemotin*, a gene encoding an 88aa transmembrane smORF peptide localised to early endosomes in *Drosophila* macrophages. *hemotin* regulates endosomal maturation during phagocytosis by repressing the cooperation of 14-3-3ζ with specific phosphatidylinositol (PI) enzymes. *hemotin* mutants accumulate undigested phagocytic material inside enlarged endo-lysosomes and as a result, *hemotin* mutants have reduced ability to fight bacteria, and hence, have severely reduced life span and resistance to infections. We identify Stannin, a peptide involved in organometallic toxicity, as the Hemotin functional homologue in vertebrates, showing that this novel regulator of phagocytic processing is widely conserved, emphasizing the significance of smORF peptides in cell biology and disease.

## Abstract 2

Hemotin, a small ORF-encoded transmembrane peptide, regulates endosomal maturation and phagocytosis in fruit fly macrophages through the adaptor protein 14-3-3ζ and phosphatidylinositol 3-phosphate. Hemotin's structure and function are conserved from flies to vertebrates.

## Author Summary

In our genomes there are millions of short open reading frames that could produce small peptides of less than 100 amino-acids if translated. These sequences have been so far disregarded, but increasing evidence supports the notion that a subset of these–termed smORFs–are translated; however the function of most of the resulting peptides remains unclear. Here we characterise *hemotin*, a smORF gene that encodes a transmembrane peptide of 88 amino-acids expressed in *Drosophila* macrophages, and we show that Hemotin localizes to early endosomes–vesicles involved in the traffic of material between the cell membrane and the cytoplasm. Macrophages found in *hemotin* mutants have enlarged and abnormal endosomes that delay digestion of phagocytosed bacteria. Accordingly, these mutant flies fight bacterial infections poorly, and die early. In humans we identify Stannin–a peptide previously involved in heavy metal toxicity–as the Hemotin homologue, and show that it shares Hemotin function in macrophages. These results identify the Hemotin-Stannin smORF peptides as regulators of phagocytosis, and suggest that they could have contributed to the ancestral origin of macrophage-like cells. This provides a strong example of a smORF conserved for hundreds of millions of years.

## Introduction

Multicellular organisms contain specialised cells in appropriate parts of the body, performing tasks that allow the formation and maintenance of a fully functional organism. This specialisation relies upon the modification of basic cellular processes, for example enhanced cytoskeletal mechanics in muscle cells, or enhanced endocytic activity in phagocytic cells [1]. At the molecular level, such modifications rely on tissue-specific gene products that regulate specific cell biology and physiology pathways. These regulators offer great promise as specific therapeutic targets, yet for many tissues we still ignore their identity and mechanism of action. Some unidentified cell regulators might be proteins whose functions have not been investigated yet; alternatively, some might be encoded by noncanonical gene products, such as peptides encoded by small Open Reading Frames (smORFs) of less than 100 amino acids.

smORFs have been largely disregarded by genome annotations and considered nonfunctional, but recently a number of ribosomal profiling and peptidomics studies have highlighted the apparent translation of hundreds of smORFs in the genomes of animals [2–4]. However, the functionality of these smORFs remains an open question, although a few smORFs have been studied and characterised functionally [5–8]; reviewed in [9,10]. Recently, we described a class of smORFs of about 80 codons long with a propensity to encode hydrophobic peptides with predicted alpha helix domains that localise to membranes and cell organelles [3]. The few examples of these smORFs with annotated function are widely expressed and involved in housekeeping processes, such as oxidative phosphorylation in mitochondria [3], but in principle these hydrophobic smORFs have the capacity to act as regulators in other membrane-based cellular processes, as the *sarcolamban/sarcolipin* smORF family of calcium signalling regulators illustrates [7,11].

Here we characterize *hemotin* (*hemo*), a tissue-specific smORF gene, which belongs to this class of smORFs, encoding an 88aa peptide with alpha-helical domains. *hemo* is expressed in hemocytes (*Drosophila* macrophages), where it regulates endosomal maturation during phagocytosis, the specific function of this histotype.

Hemocytes are the main component of the cellular branch of the insect immune system, and like vertebrate macrophages, they are professional phagocytes tasked with removing dying cells and microorganisms invading the body [12–15]. Although phagocytosis is a basic and ancestral cellular function that predates multicellularity, this function is greatly enhanced in these “professional” phagocytes. The molecular mechanisms underlying this cellular specialisation are actively studied, and have shown a surprising degree of conservation between insects and humans [16,17]. Central to phagocytosis seems to be the formation of the phagosome, a specialised endocytic vesicle containing the phagocytosed material [16], and its subsequent maturation and degradation through the endolysosomal pathway [18]. This processing requires basic endocytic components but also specific proteins and regulators, whose identity and functions are not yet fully clarified [1,16]. Furthermore, pathogenic microorganisms are often able to override this cellular defence of phagocytes by interfering with the processing and maturation of the endo-phagolysosome [19]. Here, we show that regulation of endocytic maturation by Hemotin is essential for hemocytes to digest phagocytosed bacteria effectively. Removal of *hemo* compromises the ability of the animal to clear bacteria from the body and severely reduces lifespan.

Molecularly, endosomal maturation requires “molecular labels” for the sorting of membrane vesicles into their appropriate endocytic compartments and their processing by fusion with appropriate organelles such as lysosomes. Some of these labels are provided by distinct phosphorylated states of Phosphatidylinositol (PI). In early endosomes, PI is phosphorylated to form PI(3)P (phosphatidylinositol-3-phosphate), which is required for endosomes to progress through the maturation process [18,20]. This phosphorylation step is mediated by PI(3) kinases such as class II PI3K68D (Phosphatidylinositol 3 kinase 68D) and class III Vps34 (Phosphatidylinositol 3 kinase Vps34), whereas the reverse dephosphorylation of PI(3)P is mediated by phosphatases such as Myotubularin (Mtm) [21]. Here, we show that Hemotin peptides bind and repress the adaptor protein 14-3-3ζ, and that in turn, 14-3-3ζ binds and promotes the function of PI3K68D. Thus, the *hemo* gene indirectly represses the PI3K68D-mediated labelling of early endosomes and this regulates subsequent steps of phagocytic processing.

We also observe that this regulatory mechanism is conserved across evolution. We have identified Stannin (Snn), encoded by a smORF of 88 codons, as the vertebrate homologue of Hemotin. The *snn* gene had been previously characterised as a mediator of cytotoxicity by organometallic compounds such as tri-methyl-tin (TMT) [22]. Our results show that Stannin is a functional homologue of Hemotin in fly hemocytes and mouse macrophages. Thus, we postulate that the previously unknown, nontoxic, and endogenous role of Stannin is also to modulate endosomal maturation by inhibiting the 14-3-3ζ-mediated stimulation of PI kinase function. Our results add to the body of evidence revealing the homology between the innate immune system of vertebrates and invertebrates [16,17], by identifying a new conserved member of this system with an essential pathogen-fighting activity. Hemotin/Stannin is, thus, a new example of a smORF conserved across vast evolutionary distances and fulfilling an important cellular regulatory function, suggesting that the structure-function conservation of the *sarcolamban-sarcolipin* smORF family from invertebrates to humans [7] is likely not to be an exception, but an example of a wider trend.

## Results

### 1—Identification and Characterisation of the *hemotin* Gene

**Fig 1:**
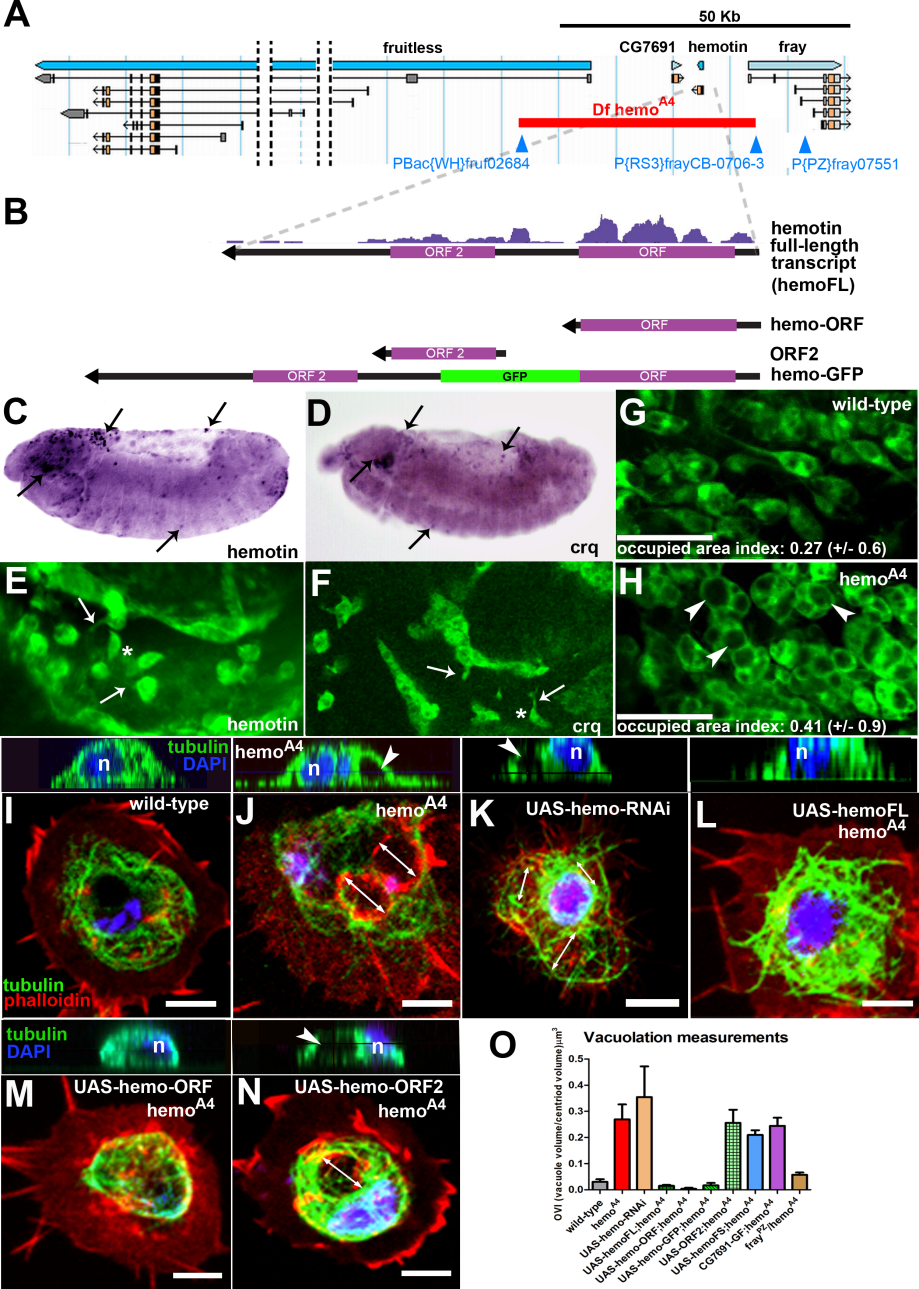
Identification and phenotypical characterisation of the *hemotin* gene. (A) *hemo* genomic locus including the *hemo*, *CG7691*, *fray*, and *fruitless* genes (blue arrows). The *hemo* ^*A4*^ deletion (red bar) was generated by FRT-mediated recombination using the *P{RS3}fray* ^*CB-0706-3*^ and the *P-Bac{WH}fru* ^*f02684*^ transposable elements (blue triangles). Transcript models are represented under their respective genes, orange boxes represent coding exons, whereas gray boxes indicate noncoding exons (untranslated regions, UTRs). *hemo* ^*A4*^ completely removes *hemo* and *CG7691* plus the first noncoding exons of *fray* and *fruitless*. The *P{PZ}fray* ^*07551*^ insertion is a lethal *fray* allele [25] and was used for genetic complementation experiments between *hemo* ^*A4*^ and *fray*. (B) Top: Ribosomal profiling reads obtained from polyribosomes from S2 cells (Poly-Riboseq;(3)) mapped to the *hemo* full-length transcript (*hemoFL*). *hemo*-ORF is translated more efficiently than ORF2 (*hemo*-ORF RPKM: 29.4, coverage: 0.9 ORF; ORF2 RPKM: 6.6, coverage: 0.7. Note that the reads per kilobase of transcript per million mapped reads [RPKM] value of ORF2 is below the 11.8 cut-off to be considered translated [3]). Bottom: schematic representation of other constructs used in this manuscript. *hemo*-ORF is a minigene consisting of an mRNA fragment truncated immediately after the *hemo*-ORF stop codon, ORF2 consists of a mini-gene construct carrying the ORF2 sequence only, including 6 nt upstream of its start codon (to conserve its endogenous Kozak sequence). *hemo*-GFP (green fluorescent protein) is a *hemo*-ORF-GFP fusion construct in which the GFP sequence (devoid of a start codon) was cloned into the *hemoFL* construct, immediately downstream and in frame with *hemo*-ORF (devoid of a stop codon) (see Materials and Methods). (C) Pattern of expression of *hemo* in germ band-retracted embryos revealed by *in situ* hybridisation. *hemo* is specifically expressed in embryonic hemocytes (arrows; compare with D) in the head, amnioserosa, and dispersed along the body. (D) Spatial distribution of embryonic hemocytes at germ band retraction stage revealed by *in situ* hybridisation of hemocyte-specific *croquemort* (*crq*) gene, showing similar distribution in the head, amnioserosa, and along the body (arrows). (E) Cluster of early embryonic hemocytes of the cephalic region expressing the *hemo* transcript revealed by FISH (fluorescent in situ hybridisation). Some hemocytes show drop-shape morphologies (asterisk) and membrane projections such as filopodia (arrows). (F) Embryonic hemocytes labelled with *crq-Gal4;UAS-GFP* expression from the head region displaying similar cellular morphologies (arrows and asterisk) as those in E. (G–H) White prepupal thoracic hemocytes revealed by *crq-Gal4;UAS-GFP* expression in wild-type (G) and *hemo* ^*A4*^ mutants (H). In *hemo* ^*A4*^ mutants, hemocytes display enlarged vacuoles within the cytoplasm (arrowheads), with larger occupied area index (OAI). Scale bar (50 μm). (I–N) hemocytes observed *ex vivo* [15] showing Tubulin (green) and Actin (red) cytoskeletons and nuclei (2-(4-Amidinophenyl)-6-indolecarbamidine dihydrochloride, DAPI) with its corresponding orthogonal projection of confocal microscopy z-stacks (above inset) showing only tubulin cytoskeleton (green) and DAPI (blue) staining in the nucleus (n). Scale bar (5 μm). (I) Wild-type hemocyte. (J) *hemo* ^*A4*^ mutant hemocyte shows large disruptions of the tubulin cytoskeleton that appear as rounded vacuoles (arrows; arrowhead in inset). (K) Knocking down the expression of *hemo* with a *UAS-hemo-RNAi* construct phenocopies the vacuolation phenotype (arrows and arrowhead in inset). (L) Expression of *hemo* full length transcript (*UAS-hemoFL*) rescues the vacuolated *hemo* ^*A4*^ phenotype. Expression of *hemo*-ORF only (M) also rescues the *hemo* ^*A4*^ mutant vacuolation. (N) Expression of ORF2 does not rescue the *hemo* ^*A4*^ mutant vacuolated phenotype (arrows and arrowhead in inset). (O) Vacuolation measurements in *ex vivo* primary pre pupal hemocytes. *hemo* ^*A4*^ mutant hemocytes show significantly higher occupied volume index (OVI) (see Materials and Methods) than wild-type. Rescue experiments show that the vacuolation phenotype is specific to the peptide encoded by *hemo*-ORF. All upstream activating sequence (UAS) constructs were driven by *crq-Gal4*. Error bars represent standard error of the mean (SEM). Statistical analysis was performed using one-way ANOVA test indicating that samples were significantly different [F(9,486) = 9.5, *p* < 0.0001]. A post hoc Bonferroni multicomparison test showed that *hemo* ^*A4*^, *UAS-hemo-RNAi*, *UAS-ORF2-hemo* ^*A4*^, *UAS-hemoFS* (expressing a *hemo* full-length transcript containing frameshifts in *hemo*-ORF and ORF2)-*hemo* ^*A4*^ and *CG7691* genomic fragment (GF)-*hemo* ^*A4*^ were significantly different than wild-type. The *UAS-hemoFL-hemo* ^*A4*^,*UAS-hemo-ORF-hemo* ^*A4*^,*UAS-hemoGFP-hemo* ^*A4*^ and *fray* ^*PZ*^ */hemo* ^*A4*^ were not significant to wild-type (*n* ≥ 24, *p* < 0.05). Supplemental data are shown in S1 Fig and S1 Data.

We originally identified *hemo* in a bioinformatics search for putative functional smORFs in the *Drosophila melanogaster* genome [23]. *hemo* encodes a putative transmembrane peptide of 88 amino acids that matches a putative peptide fragment from a proteomics study of *Drosophila* S2 cell membrane extracts (S1A and S1B Fig) [24]. *hemo* originally mapped to a region of the genome devoid of any annotated gene, but subsequently, this locus has been annotated by the *Drosophila* Genome Project, and its structure is shown in Fig 1A. A potential polycistronic RNA is expressed by this locus and contains the 88aa *hemo*-ORF and a second short ORF (Open Reading Frame) of 59 codons (ORF2), currently annotated in Flybase as *CG43194* and *CG43210*, respectively. Poly-Ribo-Seq data from *Drosophila* S2 cells shows that *hemo* transcripts are actively translated (Fig 1B) [3].

We assayed expression of *hemo* throughout development by reverse transcription polymerase chain reaction (RT-PCR) and found that transcription of *hemo* is temporally regulated, showing higher expression from prepupal to adult stages, in agreement with modEncode RNAseq data (S1 File;Flybase). However, *in situ* hybridisation also showed that *hemo* is specifically expressed in *Drosophila* embryos in a pattern similar to *crq*, a hemocyte-specific marker (Fig 1C–1F) [26–28]. Accordingly, *hemo* was detected by RT-PCR from prepupal hemocyte RNA extractions (S1C Fig). Hemocytes are fly macrophages involved in the engulfment of cellular corpses throughout development, and they are the cellular branch of the fly innate immune system [12].

To elucidate the function of *hemo*, we generated a deletion of the locus by FRT-mediated recombination using the *P(RS3)fray CB-0706-3* and *PBac(WH)fru f02684* P-element insertions [29]. This deletion (*hemo* ^*A4*^) removes the entire *hemo* locus, *CG7691*, and also one 5’ noncoding exon of each of the *frayed (fray)* and *fruitless* (*fru*) genes (Fig 1A and S1C Fig). Homozygous *hemo* ^*A4*^ mutants reached adulthood and did not show overt morphological defects; however, their hemocytes exhibited a more vacuolated morphology in comparison to wild-type controls (Fig 1G and 1H and S1D and S1E Fig). The vacuolated phenotype in *hemo* ^*A4*^ mutant hemocytes was reflected in the microtubule cytoskeleton, and the volume of the vacuoles was significantly larger than those present in wild-type hemocytes (Fig 1I, 1J and 1O).

Further genetic analysis corroborates that this hemocyte phenotype in *hemo* ^*A4*^ is specific to loss of the Hemotin peptide. The *fru* and *fray* genes have functions in dimorphic sexual behaviour and nerve fasciculation, respectively [25,30]. We discarded them as candidates for providing the requirement for hemocyte vacuolation since *fru* was not expressed in hemocytes (S1 File), and the function of *fray* is provided by a shorter transcript containing the coding exons (Fig 1A), whose expression was not affected in the *hemo* ^*A4*^ deletion (S1 File). In agreement with this, the *hemo* ^*A4*^ and *fray* ^*PZ07551*^ mutations (Fig 1A) [25] complemented each other giving rise to hemocytes with normal vacuoles (Fig 1O) and larvae with normally fasciculated nerves (S2 File). For the *CG7691* gene, we generated a 12 Kb genomic construct containing its coding and upstream regulatory regions. Hemocytes carrying this *CG7691* genomic fragment (GF) in a *hemo* ^*A4*^ mutant background expressed *CG7691* at wild-type levels (S1 File) but still possessed large vacuoles (Fig 1O), indicating that deletion of *CG7691* in *hemo* ^*A4*^ is not the cause of hemocyte vacuolation. In contrast, loss of function and rescue experiments pinpoint the *hemo* transcript as responsible for these vacuolation phenotypes. First, reduction of *hemo* RNA expression with a ds-RNA construct (*UAS-hemo-RNAi*) in hemocytes produced enlarged vacuoles mimicking the *hemo* ^*A4*^ phenotype (Fig 1K and 1O). Second, expression of the *hemo* full-length transcript rescued *hemo* ^*A4*^ phenotypes (Fig 1L and 1O), whereas expression of the same construct carrying a frame-shift in each ORF, which produces scrambled peptides, did not (Fig 1O).

Next, we assessed the contribution of each ORF to *hemo* function. Expression of UAS-minigenes containing *hemo*-ORF alone (Fig 1M and 1O) or *hemo*-ORF-GFP (green fluorescent protein-tagged peptides (*hemo*-GFP) (Fig 1O) rescued the vacuolated *hemo* ^*A4*^ phenotype, whereas *UAS-ORF2* did not (Fig 1B, 1N and 1O), indicating that the peptide encoded by *hemo*-ORF represents the functional unit of the gene in this context. Poly-Ribo-Seq data from *Drosophila* S2 cells [3] indicated high translation of *hemo*-ORF but a lower, or non-productive, rate of ORF2 translation (Fig 1B). Finally, carboxyl terminal-tagged GFP fusions of each peptide in a full-length transcript only showed expression of Hemo-GFP, which localized to intracellular membrane structures in S2 cells (S2 File) and Kc167 cells (S1F–S1F” Fig). We could only detect ORF2-GFP peptides from an ORF2-GFP fusion in a short transcript minigene that excludes *hemo*-ORF (Fig 1B; S2 File). Thus, although ORF2 does show potential for translation, altogether our results support that the 88aa transmembrane peptide encoded by *hemo*-ORF alone provides the requirement for normal vacuolation in *Drosophila* hemocytes.

### 2—Hemotin Peptides Are Required for Endolysosomal Maturation and Resistance to Infection

**Fig 2:**
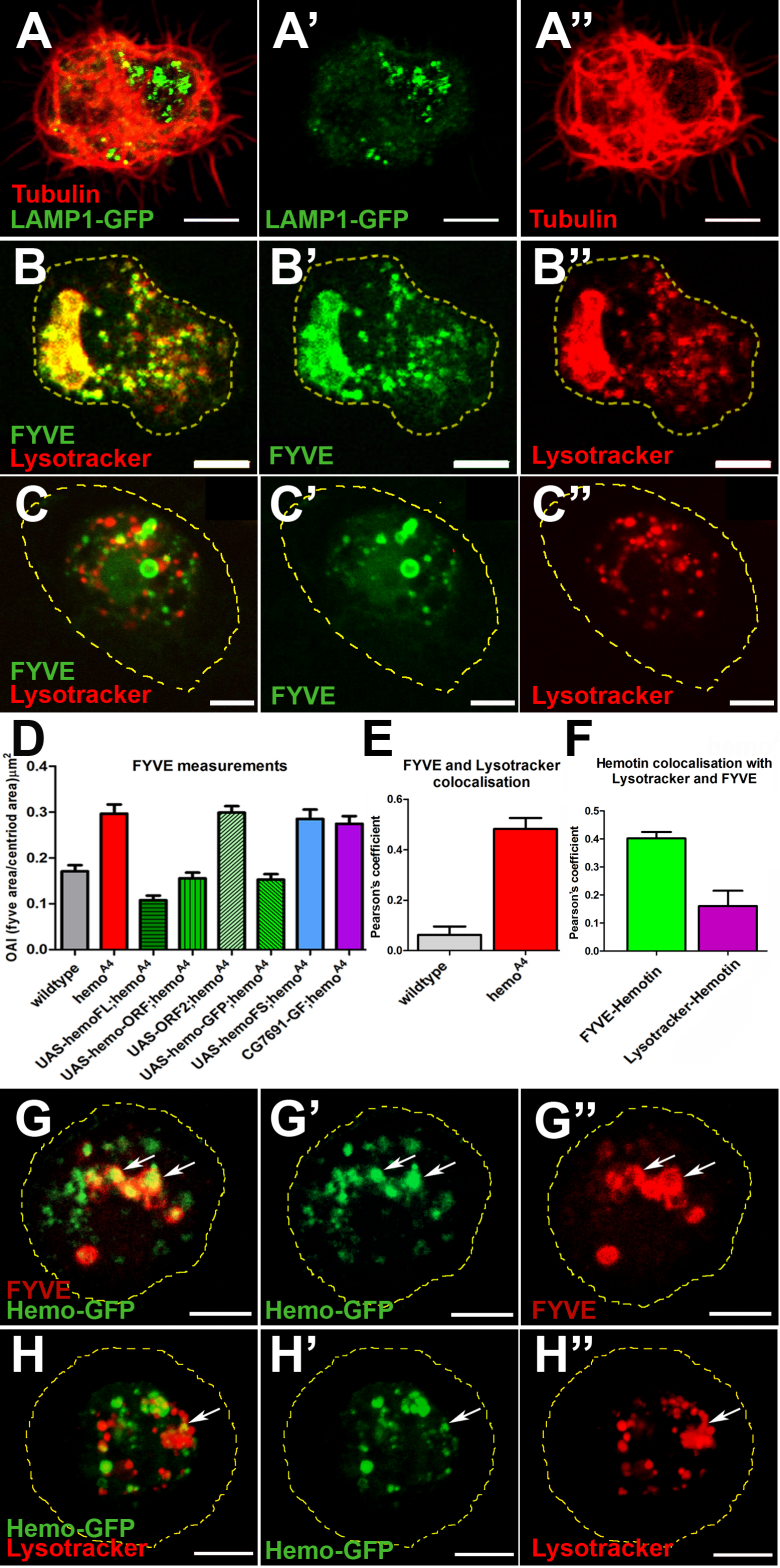
The Hemotin peptide is required for proper endosomal maturation in hemocytes. (A–A”) Distribution of acidic organelles in *hemo* ^*A4*^ mutant *ex vivo* hemocytes revealed by the expression of LAMP1-GFP lysosomal marker. The intracellular vacuoles that disrupt the beta-tubulin cytoskeleton (A, A”; red) accumulate LAMP1-GFP positive compartments (A, A’; green). Compare with wild-type in S2A–S2A” Fig. Scale bar (5 μm). (B–B”) Distribution of the endosomal marker FYVE (PI(3)P binding zinc finger domain, early endosomal marker, named after being found in Fab1, YOTP, Vac1, EEA1) (green) (B, B’) and Lysotracker (red) (B, B”) organelles in a *hemo* ^*A4*^ mutant *ex vivo* hemocyte showing enlarged intracellular compartments coexpressing FYVE and Lysotracker. Scale bar (5 μm). (C–C”) Wild-type *ex vivo* hemocyte labelled as in (B), showing little overlap between early endosome-FYVE positive (green) (C,C’) and lysosomal (red) (C,C”) compartments. (D) Quantification of the FYVE OAI in *ex vivo* hemocytes (see Materials and Methods). *hemo* ^*A4*^ mutants display a significantly larger FYVE area than wild-type. This phenotype is rescued by the expression of the *hemo* full length transcript (*UAS-hemoFL*) and is specific to *hemo*-ORF function, as it is also rescued by the expression of the *hemo*-ORF mini gene (*UAS-hemo-ORF*) or C-terminal-tagged *hemo*-GFP *(UAS-hemo-GFP*). No rescue was observed by a *CG7691* genomic fragment (*CG7691-GF*), or with a *hemo* full-length transcript containing a frameshift in the *hemo*-ORF (*UAS-hemoFS*), or with the ORF2 mini gene (*UAS-ORF2*). All UAS constructs were driven with He-Gal4. Error bars represent SEM. One-way ANOVA analysis shows that there is a statistically significant difference between these groups [F(7,286) = 27.12, *p* < 0.0001]. Post hoc comparisons using Bonferroni test indicated that the mean score of *hemo* ^*A4*^, *UAS-hemo-ORF2-hemo* ^*A4*^, *UAS-hemoFS-hemo* ^*A4*^, and *CG7691-GF-hemo* ^*A4*^ did significantly differ from wild-type (*p* < 0.05), whereas *UAS-hemoFL-hemo* ^*A4*^, *UAS-hemoORF-hemo* ^*A4*^, and *UAS-hemoGFP-hemo* ^*A4*^ did not. (E) Analysis of the overlap between FYVE-positive early endosomal and Lysotracker-positive compartments using Pearson’s correlation coefficient in wild-type and *hemo* ^*A4*^ mutant hemocytes. In *hemo* ^*A4*^ hemocytes, there exist significantly more intracellular compartments displaying FYVE and Lysotracker colocalisation than in the wild-type as shown by a Two-tailed Mann-Whitney test (*n* ≥ 17; *p* < 0.05). Error bars represent SEM. (F) Statistical analysis of the Pearson’s coefficient measurements of Hemotin-GFP (Hemo-GFP) with early endosomal (FYVE-cherry) and lysosomal (Lysotracker) markers. Tagged-Hemotin peptides are significantly enriched in early endosomal compartments in comparison with lysosomes as shown by a two tailed Mann-Whitney test (*n* ≥ 20, *p* < 0.05). Error bars represent SEM. (G–G”) Localisation of Hemo-GFP peptides (green)(G’) and the endosome FYVE marker (red)(G”) in hemocytes *(He-Gal4;UAS-hemo-GFP*). A substantial part of Hemo-GFP pattern colocalizes with FYVE-positive compartments (G) (arrows). Scale bar (5 μm). (H–H”) Distribution of Hemo-GFP peptides (green) (H’) and the lysosomal marker (lysotracker; red) (H”) in hemocytes. Only a small overlap exists between Hemo-GFP compartments and lysosomes (H) (arrow). Supplemental data are shown in S2 Fig, S6 Fig, and S1 Data.

We next investigated the origin of the mutant vacuoles. *hemo* ^*A4*^ hemocytes showed an increased accumulation of acidic compartments as revealed by pH-sensitive Lysotracker (S1G, S1H and S1K Fig), and vacuoles disrupting the microtubule cytoskeleton contained the lysosomal marker LAMP1 (Figs 2A–2A” and S2A–S2A”). Expression of *hemo* full-length or *hemo*-ORF constructs in *hemo* ^*A4*^ mutants rescued the enlarged acidic compartment phenotype (S1I–S1K Fig). These pieces of evidence suggest that the enlarged vacuoles are some type of abnormal degradation compartment and that *hemo* is necessary for the processing of phagocytic or recycled materials.

We have used further endocytic markers to ascertain the nature and integrity of the acidic compartments in *hemo* ^*A4*^ null hemocytes. The *hemo* ^*A4*^ enlarged acidic intracellular organelles show an extensive overlap of early and late endocytic markers: FYVE (a PI(3)P-binding zinc finger domain, early endosomal marker, named after being found in Fab1, YOTP, Vac1, EEA1 [31]) (Fig 2B and 2B’ and 2E and S2E and S2E’ Fig), Rab7 (late endosomal marker) (S2C–S2C’, S2E–S2E’ Fig), and Lysotracker (acidic organelle marker) (Fig 2B, 2B” and 2E and S2C and S2C” Fig) in comparison with wild-type hemocytes (Fig 2C–2C” and 2E and S2B–S2B” and S2D–S2D” Fig). This combination of early and late markers identifies the enlarged compartments as aberrant endolysosomes [21]. We quantified the occupied FYVE area index (see Materials and Methods; Fig 2D) and the average diameter of FYVE vesicles (see Materials and Methods; S6 Fig). *hemo* ^*A4*^ mutant hemocytes showed larger FYVE compartments containing larger vesicles than wild-type. Expression of *UAS-hemo* full-length transcript, *UAS-hemo-ORF*, and *UAS-hemo-GFP* constructs rescued these phenotypes, whereas expression of a *UAS-hemo frame-shift*, *UAS-ORF2* and a *CG7691 GF* constructs failed to do so (Fig 2D). The simplest interpretation of our results is that Hemotin peptides are required for completing the maturation of at least some early-to-late endosomes, and hence for their subsequent endolysosomal degradation.

Other disruptions of this traffic could also perturb endosomal maturation, since endolysosomal homeostasis depends on the balance between trafficking of membrane from the endocytic pathway (influx) and exit of membrane (efflux) into early recycling compartments [18]. We explored whether the membrane efflux to early recycling compartments was disrupted in *hemo* ^*A4*^ mutants by visualizing Rab11, a marker of recycling endosomes. In *hemo* ^*A4*^ mutant hemocytes, the size of Rab11-positive compartments seemed normal in comparison with wild-type (S2F–S2F’ and S2G–S2G’ Fig), suggesting that membrane efflux to early recycling compartments is not grossly affected. Thus, the role of Hemotin peptides seems to focus at promoting early to late endosomal maturation.

To corroborate this hypothesis, we ascertained the precise subcellular localisation of Hemotin peptides in hemocytes. The Hemo-GFP peptides, which are functional and able to rescue *hemo* ^*A4*^ mutants (Fig 1B and 1O and Fig 2D), localised to early endosomes, as shown by their colocalization with the FYVE marker (Fig 2F and 2G–2G”) and HRS proteins [32] (S2H–S2H” Fig) but only limited colocalization with the late endocytic marker Lysotracker (Fig 2F and 2H–2H”). Thus, the *in vivo* Hemotin peptide localization correlates with their inferred role in promoting endosomal maturation.

**Fig 3:**
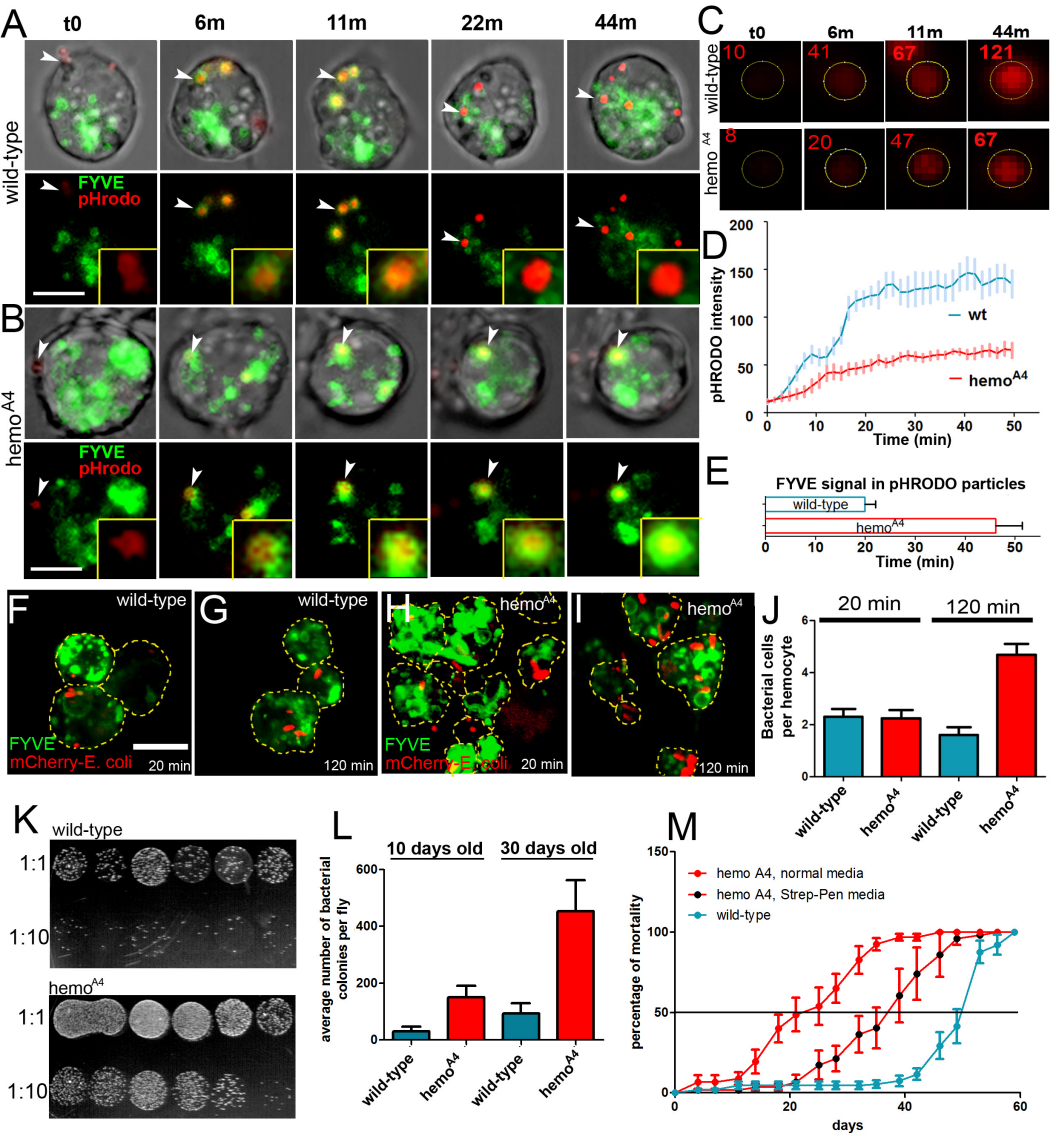
*hemotin* is involved in phagocytic processing and is necessary for optimal bacterial clearance and life span. (A–B) Time-lapse imaging showing the phagocytic trafficking of pHrodo-labelled bacterial particles (arrowhead, red) in *ex vivo* hemocytes expressing the early endosomal marker FYVE-GFP (green) with the *He-Gal4* driver in wild-type (A) or *hemo* ^*A4*^ mutants (B) (see S1 and S2 Videos). (t0) represents the time when the particle docks into the cell membrane, displaying a relatively faint intensity. By t = 6 minutes (min), the pHrodo particles are in FYVE-positive early endocytic vesicles in both wild-type (A) and *hemo* ^*A4*^ mutant hemocytes (B). By t = 22 min in the wild-type hemocyte (A), the FYVE signal around the particle is dramatically reduced, while the intensity of the pHrodo signal increases, suggesting that the vesicle has progressed into a PI3P-depeleted and acidified endolysosome. In the *hemo* ^*A4*^ mutant hemocyte (B), the FYVE signal around the particle is still visible at t = 44 min, showing an increased prevalence of PI(3)P in this vesicle, and therefore an extended early endocytic phase. However, the intensity of the pHrodo signal is lower than in wild-type, indicating a delay in the acidification of the endocytic vesicles (See also C and D). The insets show a magnification of the specific particles. Scale bars = 5 μm. (C) Magnified raw images from the particles shown in (A) and (B), showing the region of interest (ROI) used to quantify their fluorescent intensity. The integrated intensity read-out for each time point is indicated in red. (D) Quantification of fluorescent intensity of pH-sensitive pHrodo particles undergoing phagocytosis in wild-type (blue) or *hemo* ^*A4*^ mutant (red) hemocytes. Average integrated intensity per pixel is represented for each time point. Error bars represent SEM. A two-way ANOVA analysis with Bonferroni post test indicates that the difference between these curves is significantly different from t = 16.5 min (*n* = 8, *p* < 0.001). (E) Quantification of FYVE prevalence on pHrodo particles undergoing phagocytosis in wild-type (blue) or *hemo* ^*A4*^ mutant (red) hemocytes. The FYVE signal remains significantly longer in *hemo* ^*A4*^ mutants (mean = 46.06 +/− 5.3 min) than wild-type (mean = 19.87 +/− 2.1 min) as indicated by a one-tailed unpaired *t* test (*n* = 11, *p* < 0.0003). Error bars represent SEM. (F–I) Visualisation of bacterial up-take and processing *in vivo*. Dorsal vessel-associated adult hemocytes (see Materials and Methods) expressing FYVE-GFP (green) driven by *He-Gal4* from wild-type (F, G) or *hemo* ^*A4*^ mutant flies (H, I) infected with mCherry-expressing *E*. *coli* bacteria (red) (OD600 = 0.05) and dissected 20 min (F, H) or 120 min (G, I) postinjection. Wild-type and mutant flies exhibit a similar number of bacterial cells per hemocyte after 20 min; however, this number increases in mutant flies after 120 min, whereas it remains constant in wild-type flies, suggesting that mutant hemocytes accumulate undigested bacterial cells. Yellow dashed lines represent the cell body outline. (J) Quantification of bacterial uptake. *hemo* ^*A4*^ mutant hemocytes contain a similar number of bacterial cells as wild-type after 20 min but significantly more bacterial cells after 120 min. Average number of bacterial cells per hemocyte is represented in the *y*-axis. (*n* = 30, *p* < 0.0001). (K) Visualisation of bacterial load from either wild-type or *hemo* ^*A4*^ mutant 30days (d)old adult fly homogenates. Each spot represents the bacterial colonies grown from an individual male fly. Each homogenate was plated in decreasing dilution (1:1 or 1:10). Note the higher density of bacterial colonies in the spots from *hemo* ^*A4*^ mutants compared to wild-type. (L) Quantification of bacterial colonies grown from single adult fly homogenates. *hemo* ^*A4*^ mutants contain significantly more bacteria than wild-type, both at 10 (*n* = 12, *p* < 0.0054) or 30 d old (*n* = 8, *p* < 0.0078). Error bars represent SEM. (M) *hemo* ^*A4*^ mutants (red) have a reduced viability over time compared to wild-type (blue), with a median life-span of 23 +/− 6 d, compared to 49 +/− 3 d for wild-type. The addition of antibiotics (penicillin-streptomycin) to the food media significantly increases the median life span of *hemo* ^*A4*^ mutants to 38 +/− 6 d, as determined by a paired *t* test (*p* < 0.05). For each condition, five different replicates were analysed for a total of 50 flies. Error bars represent SEM. Supplemental data are shown in S3 Fig, S1 Video, S2 Video, and S1 Data.

Hemotin’s requirement in endolysosomal maturation could be related to the digestion of phagocytic materials and hence have an effect on immune defence and individual survival. We analysed *ex vivo* the ability of hemocytes to phagocytise and digest bacteria by monitoring uptake of pHrodo-*E*. *coli* bacterial particles, which fluoresce in acidic compartments such as endolysosomes, while simultaneously revealing early endosomes with the FYVE marker (Fig 3A–3E; S1 and S2 Videos). *hemo* ^*A4*^ mutant hemocytes internalised pHrodo particles at similar rate to wild-type hemocytes (S3A Fig), indicating that the initial phagocytic uptake is not affected. However, in the *hemo* mutants, the internalised particles acidified at a slower rate and to a lesser degree, and remained for longer in FYVE-positive vesicles (Fig 3A–3E), suggesting a reduced ability of *hemo* ^*A4*^ mutant hemocytes to digest phagocytosed material. Such impairment could compromise the mutant fly’s ability to deal with invading and commensal microorganisms, especially for those usually cleared through phagocytosis by hemocytes [33,34]. Indeed, *hemo* ^*A4*^ mutant hemocytes also showed *in vivo* a similar normal uptake but slower processing of intact *E*. *coli* bacteria expressing *mCherry* (Fig 3F–3J), and homozygous *hemo* ^*A4*^ mutants carried a higher bacterial load than wild-type flies raised simultaneously in the same vial (Fig 3K and 3L). This increased bacterial load seems to affect lifespan, since *hemo* mutants had a median life span that is only 47% of the wild-type (Fig 3M) in normal cultures, but this increases to 77% when raised in germ-reduced media supplemented with antibiotics (Fig 3M). Furthermore, *hemo* ^*A4*^ mutant flies show reduced resistance when infected with normally nonpathogenic *E*. *coli* bacteria, comparable to other mutants that have a reduced capacity for phagocytosis [33,34] (S3B and S3D Fig).

These results suggest that *hemo* mutants have a reduced cellular immunity. However, their humoral immunity (driven by antibacterial peptides [13]) does not seem affected. *hemo* ^*A4*^ median life span after infection with pathogenic bacteria such as *Micrococcus luteus* and *Enterobacter cloacae*, which overcome hemocytes but fully engage the humoral production of antibacterial peptides [13,33,34], was not significantly affected (S3B, S3C and S3D Fig), and accordingly the production of antibacterial peptides in *hemo* ^*A4*^ mutants was not impaired (S1 File).

Thus, our results suggest that Hemotin peptides in the early endosomes of fly macrophages are required for normal phagocytic processing and that absence of these peptides results in abnormal and slower maturation and degradation of phagocytic materials. In turn, this abnormal phagocytic processing reduces the ability of the organism to fight off bacteria and has a direct impact on fly immunity and viability.

### 3—Hemotin and Vertebrate Stannin Belong to a Conserved Family

**Fig 4:**
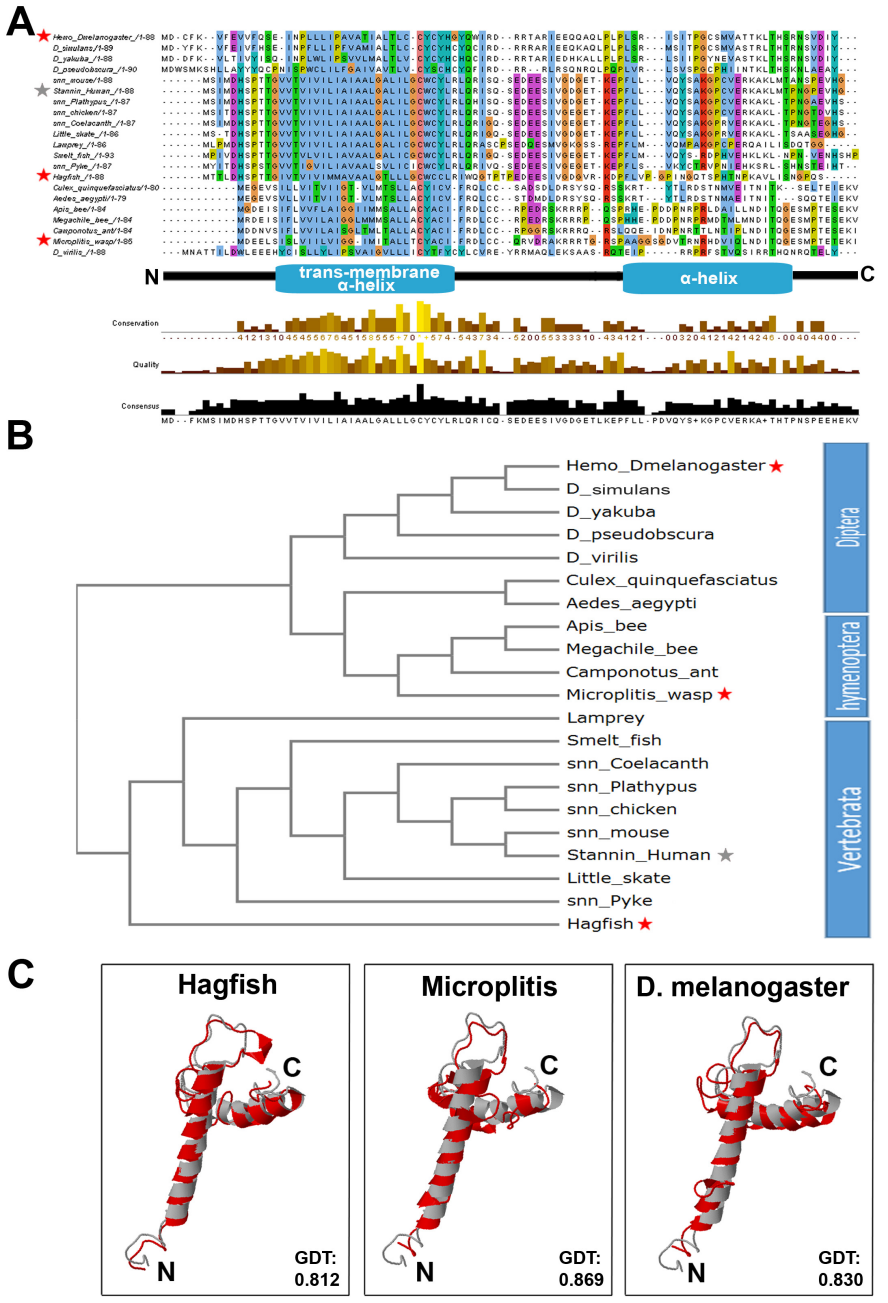
Conserved sequence and structure in Stannin, the Hemotin vertebrate homologue. (A) Alignment showing Hemotin amino acid peptide sequences (see S4 File) from two insect lineages (Diptera and Hymenoptera) and vertebrate Stannin. The “Conservation” lane at the bottom reflects the conservation of physical–chemical properties in the amino acids, while the “Quality” lane scores the likelihood of observing the mutations displayed in each particular position of the alignment. Note that there exists high conservation in the transmembrane domain (blue) motif among these peptides. The N-terminal transmembrane and C-terminal alpha-helix domains are indicated below the alignment. Sequences studied in C) (see below) are highlighted with stars. (B) Cladistic guide tree showing the relationships between insect Hemotin and vertebrate Stannin peptides shown in (A). The Hemotin and Stannin sequences cluster with their respective counterparts from related species. Insect Hemotin sequences are branched into two main clusters, one represented by flies and the other by hymenopterans, with other dipterans such as mosquitoes being between these two. New Stannin homologues identified by us cluster with annotated Stannin sequences (prefixed by snn_) and show an overall correct phylogenetic position, with agnathans Lamprey and Hagfish basal to other vertebrates and closer to insect sequences (see also S4 Fig). Sequences studied in C) (see below) are highlighted with stars. (C) Threading of the peptide sequences (shown in red) from a basal vertebrate (hagfish), a basal hymenopteran (the wasp *Microplitis)*, and *D*. *melanogaster* onto the human Stannin structure (shown in grey) (see Materials and Methods). It has been proposed that the N-terminal α-helix of the Stannin peptide transverses the membrane at an 80^o^ angle, whereas the C-terminal α-helix lies upon the lipid bilayer at the cytoplasmic side [36]. Note that the three peptides can adopt similar tertiary structures. GDT indicates the value of the High-Accuracy Global Distance Test, which measures the average distance (in Angstroms) between the Human Stannin model and the model for each peptide [38]. As expected, the Hagfish peptide, more closely related to human Stannin, obtains a lower distance, but insect peptides display values in the same range despite their lower sequence similarity (see A) above and S4C Fig). Supplemental data are shown in S4 Fig, S3 File and S4 File.

To elucidate the molecular function of the Hemotin peptide, we searched for similarity to known structural domains using the Phyre2 engine [35]. The human Stannin peptide is encoded by an 88aa smORF-like Hemotin and appeared as a possible match (Fig 4A). Structural analyses of the Stannin peptide suggest a transmembrane peptide containing two alpha helices, an N-terminal one that spans the lipid bilayer and a C-terminal helix at the cytosolic side (Fig 4A) [22,36], and our independent analysis of the Hemotin sequence using a transmembrane topology prediction program [37] also revealed a very similar potential transmembrane alpha-helical domain (Fig 4A, S1B Fig). Threading the Hemotin sequence onto the predicted human Stannin tertiary structure (see Materials and Methods) confirms a structural compatibility similar to vertebrate members of the Stannin family (Fig 4C).

We further searched for homologues of Hemotin and of Stannin following a bioinformatics pipeline used in Magny et al., 2013 [7]. We identified homologues of Hemotin peptides in other dipterans (mosquitoes) and in other insects such as hymenopterans (bees, ants, and wasps), plus new homologues of Stannin in ancestral vertebrates, such as hagfish, lamprey, and cartilaginous fishes (Fig 4A and 4B and S4A and S4B Fig), and these sequences also show structural compatibility with Stannin (Fig 4C and S4C Fig). Despite considerable amino acid sequence variation, comparison of Hemotin and Stannin sequences reveals conservation in the alpha-helices and in the intervening sequence, which have been implicated in Stannin function [22] (Fig 4A). The resulting tree of Stannin and Hemotin sequences shows a good correlation with the animal phylogeny (Fig 4B) and correctly locates the 88aa mitochondrial ribosomal protein S21 as an outgroup (S4B Fig). Altogether, the amino acid sequence analyses suggest that that Stannin and Hemotin are members of the same peptide family, displaying sequence and structural homology.

### 4—Hemotin and Stannin Are Functional Homologues That Interact with 14-3-3ζ

Stannin is a peptide involved in organometallic toxicity, but its endogenous physiological and cellular functions have remained elusive [22]. In rodent models, *snn* expression has been detected in hematopoietic organs and immune cells including macrophages [39–41]. Similarly as with Hemotin (see above; unp. obs.), Flag-tagged Snn peptides have been detected in membrane fractions of intracellular compartments, such as endoplasmic reticulum, peroxisomes, mitochondria, and endosomes in mouse NIH-3T3 cell lines [42].

**Fig 5:**
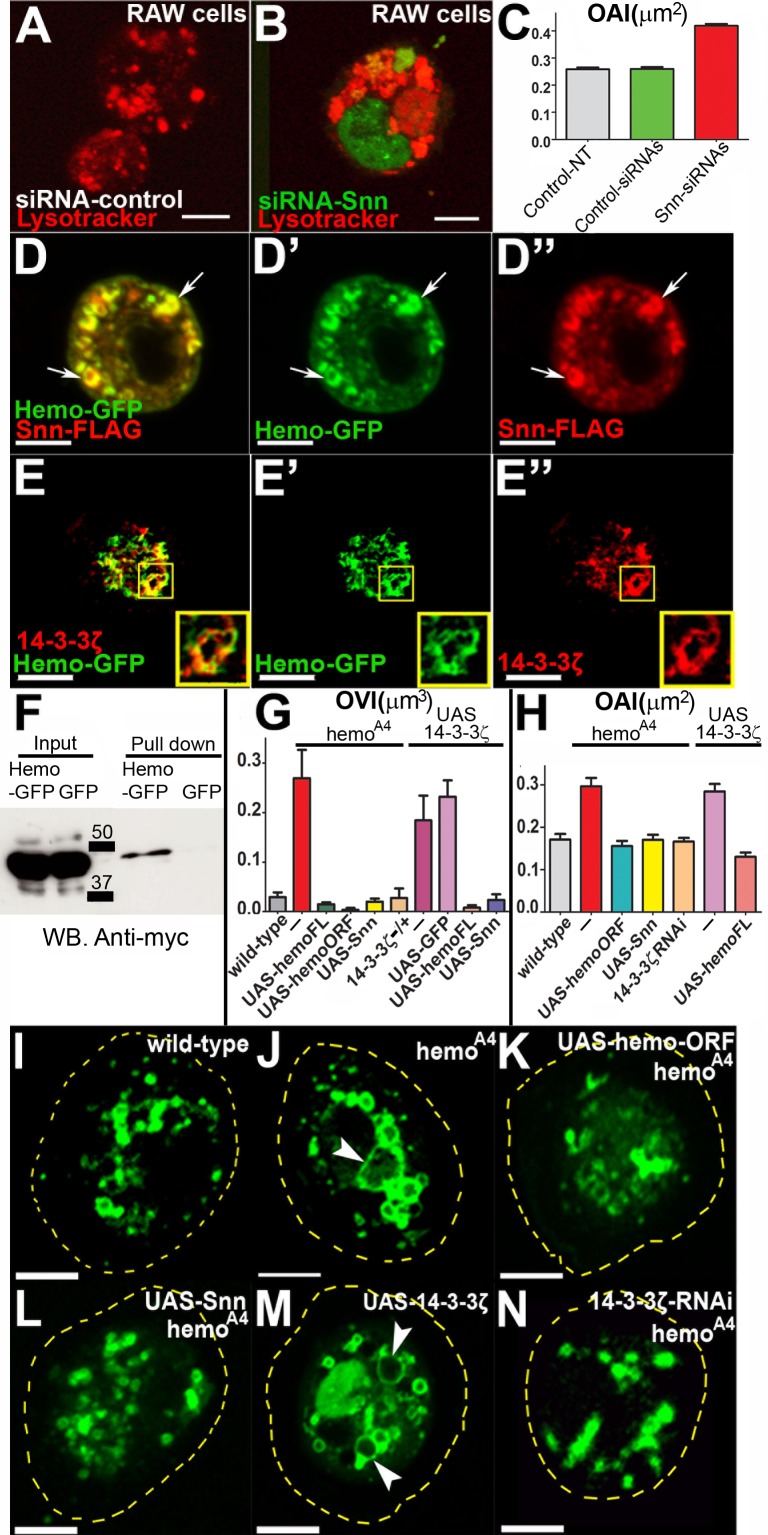
Stannin and Hemotin replicate each other’s functions and counteract 14-3-3ζ function during endosomal maturation. (A) Mouse macrophage-like RAW264.7 cells treated with control-scrambled siRNAs and labelled with the acidic pH-sensitive Lysotracker (Red). Scale bar (5 μm). (B) RAW264.7 cells treated with two Fluorescein isothiocyanate (FITC)-labelled *snn* siRNAs (green) (see S5 Fig) and stained with Lysotracker. Note the highly enlarged lysosomal compartment in *snn* siRNA-treated cells (see C). Scale bar (5 μm). (C) Lysosomal OAI, revealed by Lysotracker, in nontransfected or control siRNA and *snn* siRNA-treated RAW264.7 cells, showing that *snn* siRNA-treated RAW 264.7 cells have significantly larger lysosomal compartments than control samples (see S1 Data). The graph shows averages of three independent experiments. One-way ANOVA test showed that samples were significantly different [F(2,780) = 185.6, *p* < 0.0001]. Post hoc Bonferroni multicomparison test showed that the siRNA-*snn* sample was significantly different to nontransfected and siRNA-control samples (*n* ≥ 240, *p* < 0.05). On average, *snn* siRNA-treated cells show a reduction in *snn* expression of 60% relative to nontreated cells, whereas cells treated with control siRNA only show a reduction of 0.8% (see S5A Fig). Error bars represent SEM. (D–D”) Hemo-GFP and Snn-FLAG peptides expressed in hemocyte-like *Drosophila* Kc165 cells (20) using the *Act5-Gal4* driver. Hemo-GFP (green) (D’) and Snn-FLAG (red) (D”) peptides colocalize in intracellular vesicles and punctate organelles (arrows). Scale bar (5 μm). (E) Colocalisation of Hemo-GFP peptides and Nt-tagged HA-14-3-3ζ protein in intracellular compartments in *ex-vivo* hemocytes. (E’) Hemo-GFP. (E”) HA-14-3-3ζ. (E”‘) Merged image. UAS constructs were driven with *He-Gal4*. Scale bar (10 μm). (F) Pull down of myc-14-3-3ζ with Hemo-GFP in transfected *Drosophila* Kc167 cells. Myc-14-3-3ζ interacts with Hemo-GFP but not with a GFP-only control. Molecular weight is indicated in kilodaltons Retention of Hemo-GFP and GFP is shown in S5C Fig. (G) Vacuole OVI measurements (see Materials and Methods and S1 Data) in primary hemocytes. Expression of human *snn* (*UAS-snn*) rescues the *hemo* ^*A4*^ vacuolation phenotype to a similar extent as the rescue observed by the *UAS-hemoFL* and *hemo-ORF* constructs. Reducing the dosage of *14-3-3ζ* (in a heterozygous null *14-3-3ζ* ^*12BL*^ */+* background, labelled *14-3-3ζ −/+*) reduces the *hemo* ^*A4*^ vacuolation phenotype. Conversely, overexpression of *14-3-3ζ* (*UAS-14-3-3ζ*) in hemocytes induces the formation of larger vacuoles. The induction of vacuoles by excessive 14-3-3ζ is reversed by simultaneous overexpression of *hemo* full-length transcript (*UAS-hemoFL*) or overexpression of the human Stannin peptide (*UAS-snn*) but not by the expression of the control *UAS-GFP* construct. One-way ANOVA test showed that the means were significantly different [F(9,365) = 14.26, *p* < 0.0001]. Post hoc multiple comparison Bonferroni’s test showed that *hemo* ^*A4*^, *UAS-14-3-3ζ* and *UAS-14-3-3ζ;UAS-GFP* samples were significantly different than wild-type, whereas the rest were not (*n* ≥ 20, *p* < 0.05). Error bars represent SEM. (H) Measurement of the occupied FYVE area index (OAI) in *ex vivo* prepupal hemocytes (see Materials and Methods and S1 Data). Overexpression of human Snn peptide (*UAS-snn*) rescues the *hemo* ^*A4*^-enlarged FYVE compartments. Similarly, reducing *14-3-3ζ* function by expression *14-3-3ζ-RNAi* restores the size of *hemo* ^*A4*^ mutant FYVE-organelles to wild-type. Conversely, overexpression of *14-3-3ζ* (*UAS-14-3-3ζ*) mimics the *hemo* ^*A4*^ mutant FYVE phenotype. The overexpression *14-3-3ζ*-phenotype is reversed by coexpression with *hemo* full-length transcript (*UAS-hemoFL*). UAS constructs were driven with *He-Gal4*. One-way ANOVA test showed that the means of samples were significantly different [F(8,346) = 23.15, *p* < 0.0001]. Multiple comparison post hoc Bonferroni’s test indicated that *UAS-14-3-3ζ* and *hemo* ^*A4*^ were significantly different than wild-type whereas the rest of the samples were not (*n* ≥ 20, *p* < 0.05). Error bars represent SEM. (I–N) Intracellular distribution of FYVE-positive (green) compartments in *ex vivo* prepupal hemocytes (see also H). Scale bar (5 μm). (I) In wild-type FYVE-positive organelles appear as small rings and punctae. (J) In *hemo* ^*A4*^ mutant hemocytes, FYVE compartments contain larger rings than wild-type (arrowheads). (K) Expression of Hemo-ORF peptide (*UAS-hemo-ORF*) rescues the enlarged *hemo* ^*A4*^ mutant FYVE compartments. (L) Expression of *snn* (*UAS-snn*) reduces the *hemo* ^*A4*^ FYVE phenotype. (M) Overexpression of *14-3-3ζ* (*UAS-14-3-3ζ*) produces enlarged FYVE compartments (arrowheads). (N) Reducing *14-3-3ζ* function with RNAi rescues the *hemo* ^*A4*^ mutant FYVE phenotype. UAS constructs were driven with *He-Gal4*. Yellow dashed lines indicate the cell body. Supplemental data are shown in S5 Fig, S6 Fig, and S1 Data.

To explore the endogenous cellular function of *hemo’s* vertebrate relative, *snn*, in a vertebrate innate immune cell context, we used mouse RAW264.7 (macrophage-like) cells [43]. Firstly, we confirmed expression of *snn* in this cell line (S5A and S5B Fig). Secondly, we knocked down the expression of *snn* in RAW264.7 cells using siRNAs (small interfering RNAs) (S5A and S5B Fig) and monitored the formation of acidic compartments with Lysotracker. *snn* si-RNA-treated RAW 264.7 cells showed abnormally large acidic aggregates (Fig 5A–5C) mimicking the *hemo* ^*A4*^ null phenotype observed in hemocytes (Fig 2A” and 2L” and S1G–S1K Fig). Expressing a GFP-tagged human Stannin (Snn-GFP) peptide in *Drosophila* Kc167 cells revealed its localisation to cellular membranes, comprising intracellular punctate compartments (S5D–S5D” Fig). In addition, coexpression of tagged Stannin and Hemotin peptides in these cells show their colocalisation in membrane intracellular organelles (Fig 5D–5D”). These results suggest that Stannin and Hemotin peptides localize to similar cellular compartments and are involved in the formation of acidic compartments, suggesting a functional homology. To test this possibility, we measured the ability of Stannin to rescue *hemo* ^*A4*^ mutant phenotypes in hemocytes: expression of the human Stannin peptide rescued the vacuolated phenotype (Fig 5G) and the size of the FYVE-positive vesicles (Fig 5H, 5I, 5J and 5L; S6 Fig) of *hemo* ^*A4*^ mutants almost as effectively as endogenous Hemotin peptides (Fig 5G, 5H, 5I, 5J and 5K; S6 Fig), supporting that *hemo* and *snn* are functional homologues.

Stannin peptides bind to the 14-3-3ζ adaptor protein, although the molecular and cellular implications of this binding have not been fully clarified [44]. 14-3-3ζ proteins form dimers that bind to phosphorylated amino acid residues of target proteins and modulate their functions [45]. We investigated whether a similar interaction exists between Hemotin and 14-3-3ζ during endosomal maturation in hemocytes. We observed that Hemo-GFP peptides (which localize to early endosomes, Fig 2G–2G” and S2H–S2H” Fig) colocalised with 14-3-3ζ in hemocytes (Fig 5E–5E”‘). In addition, Hemo-GFP peptides coimmunoprecipitated with Nt-tagged 14-3-3ζ proteins expressed in *Drosophila* Kc167 culture cells (Fig 5F and S5C Fig) and hemocytes (S1 File). The coimmunoprecipitation appears moderate but reproducible and specific, perhaps reflecting a moderate affinity, or a limited amount of the multifunctional 14-3-3ζ protein [45] being engaged by Hemotin peptides.

Our genetic analysis reveals a negative functional relationship between *14-3-3ζ* and *hemo/snn* during endosomal maturation in hemocytes. Thus, excess of *14-3-3ζ* function (in *He-Gal4;UAS-14-3-3ζ* hemocytes) resembled loss of *hemo* function (as in *hemo* ^*A4*^) by producing large vacuoles (Fig 5G) and FYVE vesicles (Fig 5H, 5I, 5J and 5M), thus suggesting that *14-3-3ζ* and *hemo* work in opposite directions. An antagonistic, yet closely related, function is further suggested by gene dosage interactions. First, the *14-3-3ζ* gain of function phenotypes were corrected by simultaneous gain of either *hemo* or *snn*: both *UAS-hemo* and *UAS-snn* corrected the extra vacuolation of *UAS-14-3-3ζ* (Fig 5G), while *UAS-hemo* corrected the large FYVE vesicles produced by *UAS-14-3-3ζ*. Second, reducing *14-3-3ζ* function rescued the *hemo* ^*A4*^ phenotype to near wild-type, as indicated by vacuolation (after removing a copy of *14-3-3ζ* in a *hemo* ^*A4*^ homozygous background, Fig 5G) and the size of FYVE vesicles (by expressing *14-3-3ζ RNAi* in a *hemo* ^*A4*^ homozygous background, Fig 5J and 5N). These negative dosage interactions suggest that *14-3-3ζ* works in a common pathway, yet antagonistically, with *hemo* and *snn* [46,47]. Because *14-3-3ζ* dosage is able to modify *hemo* ^*A4*^ null phenotypes, the formal interpretation is that 14-3-3ζ acts downstream of *hemo*, or in other words, that Hemotin regulates 14-3-3ζ. Altogether, these genetic results suggest that Hemotin function represses or down-regulates 14-3-3ζ activity.

Thus, our results suggest that Hemotin and Stannin are functional homologues that are required at the cellular level for endosomal maturation; and at the molecular level, to bind and antagonize 14-3-3ζ.

### 5—Hemotin Represses the 14-3-3ζ-Mediated Stimulation of the Class II PI3K68D Kinase

**Fig 6:**
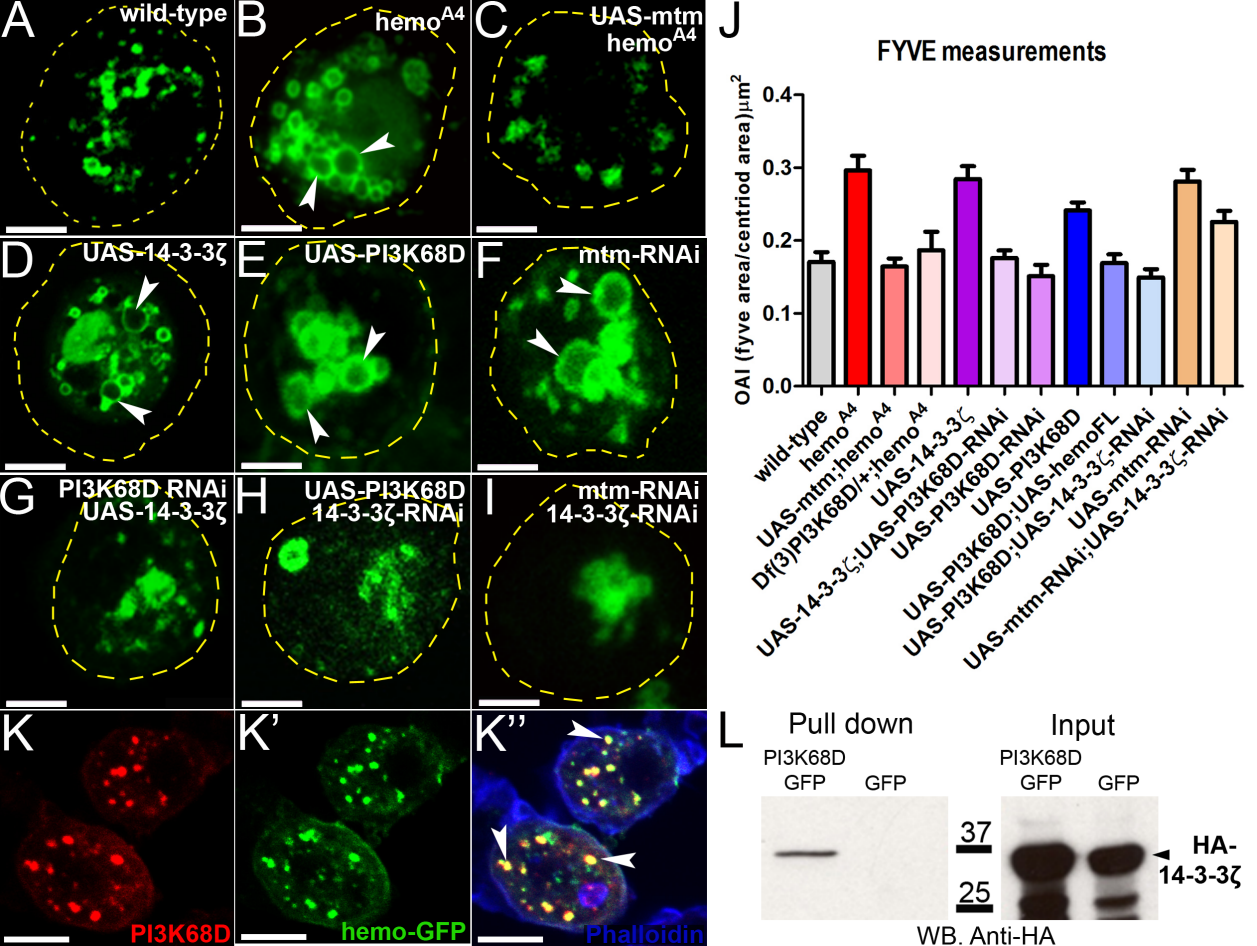
Hemotin modulates PI(3)P formation by repressing 14-3-3ζ-mediated activation of PI(3)K68D kinase. (A–I) Distribution of FYVE-positive compartments in *ex vivo* hemocytes (see also J). Yellow dashed lines indicate cell body area, and arrowheads indicate enlarged FYVE compartments. Scale bar (5 μm). (A) Wild-type hemocytes. (B) *hemo* ^*A4*^ mutants show enlarged FYVE compartments (arrowheads), (C) this phenotype is rescued by *mtm* gain of function (*UAS-mtm*). (D) Hemocytes overexpressing *14-3-3ζ* (*UAS-14-3-3ζ*) or (E) the *PI3K68D* Kinase (*UAS-PI3K68D*) also show enlarged FYVE compartments (arrowheads), similar to those observed in *hemo* ^*A4*^ mutants. (F) Enlarged FYVE compartments are also observed by the reduction of *mtm* (*mtm-RNAi*) in hemocytes. (G) Reduction of *PI3K68D* kinase function by RNAi rescues the enlarged-FYVE phenotype produced by overexpression of *14-3-3ζ*. (H) Reducing the function of *14-3-3ζ* (*14-3-3ζ-RNAi*) rescues the enlarged FYVE compartment produced by overexpression of *PI3k68D* or (I) by reduction of *mtm*. (J) Quantification of the FYVE OAI in primary hemocytes (see Fig 6, S1 Data). Knocking down the *mtm* function (mtm-RNAi) produces enlarged FYVE compartments as shown with *hemo* ^*A4*^ mutants. Both overexpression of *mtm* (*UAS-mtm*) and haploinsufficiency of *PI3K68D* (*Df(3)PI3K68D*/+), rescue the *hemo* ^*A4*^ mutant FYVE phenotype. Overexpression of *PI3K68D* (*UAS-PI3K68D*) mimics the *hemo* ^*A4*^ mutant FYVE phenotype, and this is rescued by overexpression of *hemo* full-length transcript (*UAS-hemoFL*). Reduction of *14-3-3ζ* function (*14-3-3ζ-RNAi*) corrects the *mtm* loss of function and *PI3K68D* gain of function FYVE phenotypes. The enlarged FYVE compartments produced by over-expression of *14-3-3ζ* (*UAS-14-3-3ζ*) are corrected by knocking down *PI3K68D* function (*PI3K68D-RNAi*). One-way ANOVA test indicated that means of samples are significantly different [F(12,508) = 14.01, *p* < 0.0001]. Post hoc Bonferroni’s multiple comparison test showed that *hemo* ^*A4*^, *UAS-14-3-3ζ*, *UAS-mtm-RNAi*, *UAS-PI3K68D* were significantly different to wild-type, whereas the rest of genotypes were not (*n* ≥ 19, *p* < 0.005). Error bars represent SEM. (K) In hemocytes, Hemo-GFP peptides colocalize with PI3K68D Kinase in intracellular compartments (arrowheads), presumably early endosomes. (K) PI3K68D-Cherry expression. (K’) Hemo-GFP, (K”) merge image. Scale bar (10 μm). (L) Western Blot of a Pull down experiment from hemocytes expressing PI3K68D-GFP and HA-14-3-3ζ revealing a protein interaction between PI3K68D and 14-3-3ζ. Supplemental data are shown in S6 Fig and S1 Data.

Specific steps in the endosomal maturation process depend on the phosphorylation states of PI [18,20]. In hemocytes, regulation of PI(3)P endocytic pools is key for early endosomal trafficking [21,48]. It has been shown that PI(3)P homeostasis depends on the class II PI(3)Kinase, PI3K68D, which phosphorylates PI at the carbon 3 position to form PI(3)P and the Mtm phosphatase that dephosphorylates this residue to revert to PI [21]. Either mutations in *mtm* or overexpression of *PI3K68D* increase PI(3)P in early endosomes and induce the formation of abnormal enlarged endolysosomal compartments retaining larger amounts of the PI(3)P sensor FYVE-GFP (Fig 6E, 6F and 6J) [21], similar to that observed in *hemo* ^*A4*^ mutants (Fig 2B–2B”, S2C–S2C” and S2E–S2E” Fig).

The similarities of the cellular phenotypes between *hemo* ^*A4*^ mutants and overproduction of PI(3)P by PI(3)P enzymes prompted us to carry out a genetic analysis of their functional interactions during endosomal maturation in hemocytes. The *PI3K68D* gain of function phenotype of enlarged FYVE organelles and vesicles was suppressed by coexpression of either a *hemo* full-length transcript (Fig 6J and S6 Fig) or a *14-3-3ζ-RNAi* construct (Fig 6H and 6J and S6 Fig). Similarly, reduction of *mtm* function by expressing an *mtm*-*RNAi* construct produced aberrant enlarged FYVE compartments with larger-sized vesicles (Fig 6F and 6J; S6 Fig) [21]. Codepletion of *mtm* and *14-3-3ζ* with RNAi constructs corrected the size of these abnormal FYVE compartments and vesicles (Fig 6I and 6J and S6 Fig). Formally, these results indicate that 14-3-3ζ cooperates with PI3K68D, whereas Hemotin and Mtm antagonize their action.

Three further results suggest that *mtm* and *PI3K68D* act downstream of Hemotin and 14-3-3ζ. Either overexpression of *mtm* or removing a copy of the *PI3K68D* corrected the abnormally large FYVE vesicles of *hemo* ^*A4*^ null hemocytes (Fig 6A–6C and 6J and S6 Fig). Similarly, reducing *PI3K68D* function with a RNAi construct produces little or no phenotype on its own (Fig 6J) [21] but is also able to rescue the enlarged size of FYVE vesicles produced by overexpression of 14-3-3ζ (Fig 6G and S6 Fig). Thus, in three different genetic conditions, a reduction of PI(3)P synthesis was able to suppress the enlarged and abnormal early-endosome-like vesicles produced by total loss of *hemo* or gain of function of *14-3-3ζ*. These epistatic results strongly suggest that *mtm* and *PI3K68D* act downstream of Hemotin and 14-3-3ζ during endosomal maturation; in other words, that Hemotin and 14-3-3ζ fulfil their roles through regulation of the PI(3)P enzymes.

**Fig 7:**
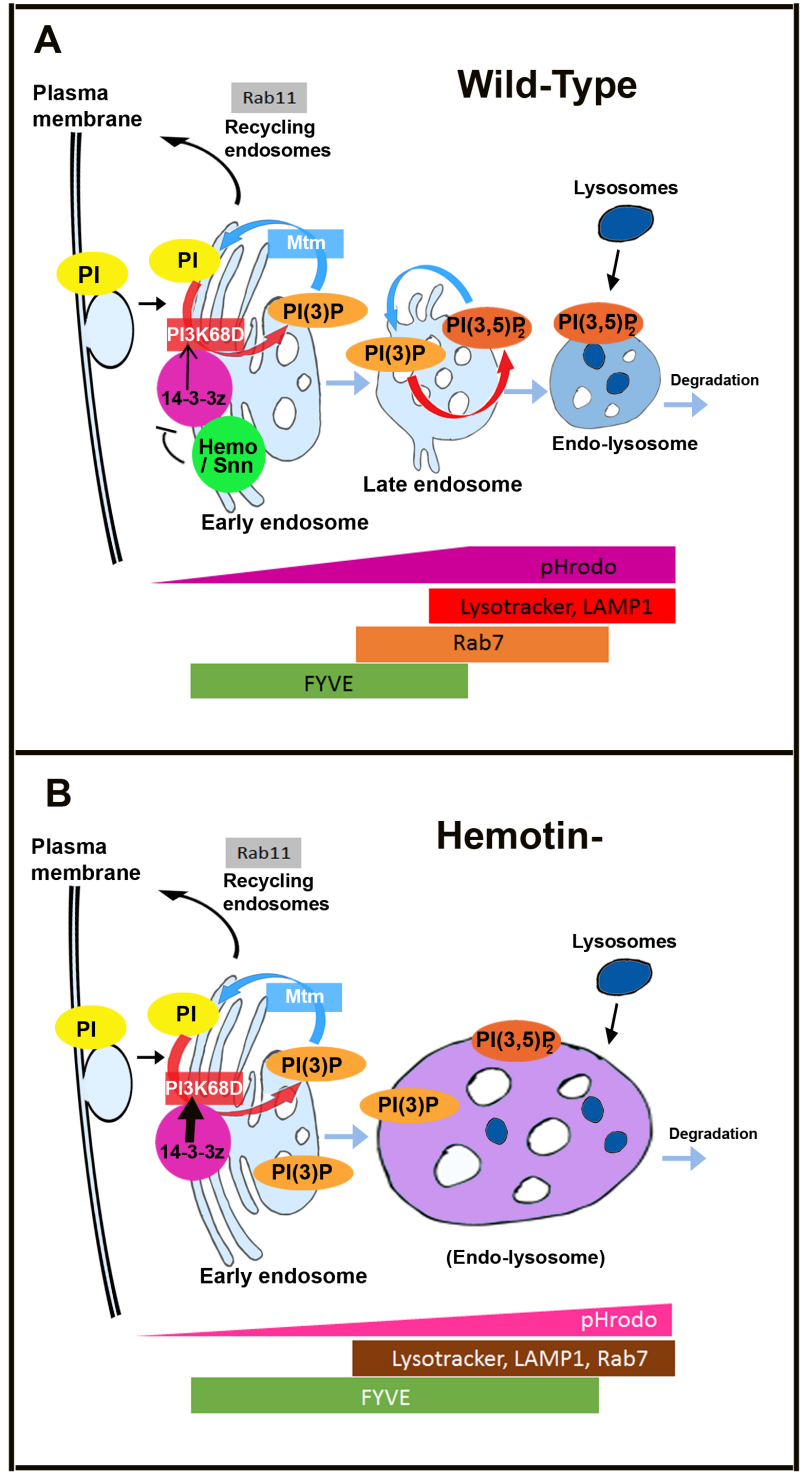
Model for the role of Hemotin in phagocytic processing. Simplified models of endosomal maturation, modified from [18] depicting the role of the proteins and markers analysed in this work. A) Wild-type endosomal trafficking is regulated by different phosphorylation states of PI. Phosphorylation of PI into PI(3)P is achieved in early endosomes by the class II or class III PI3 kinases PI3k68D and Vps34, respectively. In late endosomes PI(3)P is again phosphorylated to produce PI(3,5)P2. This phosphorylation step allows late endosomes to progress into degradation allowing lysosomes to fuse to late endosomes to produce multivesicular bodies. This trafficking progression can be reversed by dephosphorylation of PI(3)P or PI(3,5)P2 by myotubulurin phosphatases. Hemotin and Stannin are functional homologues that localise to early endosomes, where they bind and repress 14-3-3ζ. Our genetic and biochemical data indicates that 14-3-3ζ binds the PI3K68D kinase and promotes its function, perhaps by directly increasing its enzymatic activity, or indirectly by promoting its correct localisation in early endosomes. Since Hemotin antagonises 14-3-3ζ, it indirectly reduces the development of early endosomes through PI3K68D. B) The absence of Hemotin produces an excess of 14-3-3ζ function, which results in an excess of PI3K68D function and leads to an increase in endocytic vesicles containing PI(3)P, as detected by expansion of the area occupied by the FYVE marker. These abnormal vesicles display an abnormal maturation during phagocytosis, with excessive co-expression of early lysosomal markers (such as FYVE) and late ones (Lysotracker and Rab7), and a slower and less intense acidification of their contents, as revealed by the pHrodo pH marker.

Two independent lines of evidence corroborate this hypothesis. First, Hemo-GFP peptides colocalize with PI3K68D kinase at early endosomes (Fig 6K–6K”‘). Second, PI3K68D is able to pull down 14-3-3ζ from hemocyte protein extracts (Fig 6L), suggesting that 14-3-3ζ directly binds PI3K68D. Altogether, the genetic, cellular, and biochemical results support a model where Hemotin peptides indirectly affect the PI(3)P labelling of early endosomes by binding 14-3-3ζ, and hence repressing the positive effect of 14-3-3ζ on PI3K68D kinase (Fig 7).

## Discussion

Hemocytes are part of the innate immune surveillance system in *Drosophila* and are involved in the uptake of cell corpses during development and in overriding bacterial infections throughout the life cycle of the fly [12,49]. Their ease of detection and observation, coupled with the arsenal of *Drosophila* genetic techniques, makes hemocytes an excellent model system in which to characterize at molecular level basic cellular processes shared by other metazoan cells. For example, insect hemocytes display functions similar to vertebrate macrophages, such as directed migration and phagocytosis [12–15]. Similarities in the molecular mechanisms controlling these processes have been noted before [16,43,50], but the question of whether hemocytes are truly homologous to vertebrate white blood cells, representing a kind of ancestral macrophage-like cell, is not settled. Homology is supported by the clear similarities in the recognition of exogenous microorganisms through pattern recognition receptors of the Toll and Imd signalling pathways [17,51,52]. Our work further supports such homology through the identification of a modulator of endosomal maturation that is specifically expressed in hemocytes and vertebrate white blood cells and is essential for their phagocytic activity. This modulator is the Hemotin-Stannin peptide.

During endosomal maturation, turnover of PI(3)P pools at endocytic compartments is essential for PI(3)P-mediated recruiting of effectors involved in sorting specific cargo proteins and endosomal vesicles to specific trafficking routes (Fig 7A) [18,20]. In hemocytes, PI(3)P synthesis at early endosomes depends on a scaffold complex comprising both PI(3)P-turnover enzymes, PI3K68D kinase, and Mtm phosphatase bridged by Sbf (an Mtm-pseudophosphatase), indicating that PI(3)P synthesis must be tightly regulated and spatially linked to the traffic of membrane vesicles through the endosomal pathway towards degradation [21,50]. It makes sense that the function of these complexes must be tightly controlled in cell types involved in intense phagocytosis, such as hemocytes, in order to regulate PI(3)P-turnover and maintain endosomal homeostasis under the stress of enhanced membrane influx. Our results show that Hemotin peptides are specifically expressed in hemocytes and locate to early endosomes, where they bind and repress 14-3-3ζ activity. In turn, the genetic and biochemical results suggest that Hemotin-Stannin peptides prevent 14-3-3ζ from assisting the class II PI3K68D kinase at these endocytic vesicles. While the genetic suppression of the *hemo* phenotype by 14-3-3ζ and PI3K68D indicate the functional link with these proteins, this could be based on direct or indirect effects. Our ability to co-IP the two proteins is consistent with a direct effect. However, further studies are needed to confirm this and to determine how Hemotin and 14-3-3ζ can together regulate PI3Kinase activity. Thus, our results indicate that 14-3-3ζ binds PI3K68D, but do not reveal the molecular function of this binding, nor the nature of its positive impact. 14-3-3ζ could either directly stimulate the enzymatic activity of PI3K68D or promote the recruitment or retention of PI3K68D at the Sbf complex. In any case, removal of Hemotin or Mtm, or excess of either 14-3-3ζ or PI3K68D result in large PI(3)P-labelled vesicles (as revealed by the PI(3)P sensor FYVE-GFP, Figs 2 and 5), as observed in other systems when PI(3)P kinase function is deregulated [18,21]. These enlarged PI(3)P vesicles display mixed endosomal-lysosomal characteristics, and thus the normal transitory overlap of endosomal and lysosomal markers in the cell appears increased (Fig 2, S2 Fig, Fig 7B). These enlarged intermediate vesicles could occur when the increase in early endosomal vesicle formation overpowers the ability of the subsequent endosomal machinery to complete their maturation into proper endolysosomes. These mutant endosomal vesicles seem to have an impaired ability to process phagocytic material, which displays slower and less acute acidification and longer trafficking times (Fig 3). The PI(3)P-rich nature of the mutant vesicles might preclude the formation of proper endo-lysosomes, but the process of endolysosomal acidification and maturation is still not completely understood [18,53]. We propose that the cellular role of Hemotin is to reduce the endosomal PI(3)P labelling produced by the 14-3-3ζ-PI3K68D-Sbf-Mtm complex, and hence to reduce production of vesicles with early endosome characteristics (Fig 7). This modulation by Hemotin would allow for the subsequent maturation and degradation part of the endophagocytic cycle to keep up with the intake of phagocytic material, which is unaffected in *hemo* mutants.

This “endocytic modulation” appears essential in cells with high phagocytic activity such as hemocytes and macrophages to ensure proper digestion of phagocytised bacteria. In the absence of *hemo*, commensal and invading bacteria are less efficiently cleared from the body, and this ultimately compromises the viability of the organism (Fig 3, S3 Fig). Interestingly, much of this effect can be attributed to bacteria that are commensal or else not normally pathogenic, which are normally kept in check by the hemocytes, but that when this cellular defence is compromised, they overgrow in the body and lead to death (Fig 3, S3 Fig) [33]. This has interesting parallels with the severe effects of infection by normally mildly pathogenic bacteria in human patients with immunodeficiencies [54].

Regulation of endosomal maturation by Hemotin-Stannin appears to be a conserved mechanism. We have identified Hemotin homologues in other insects, and our phylogenetic analyses indicates that Hemotin is a member of a conserved peptide family, including the vertebrate Stannin, encoding peptides with similar tertiary structure (Fig 4, S4 Fig). In addition, we have demonstrated that Hemotin and Stannin are functional homologues in hemocytes during endosomal maturation, a role that appears to be conserved in vertebrates, as *hemo* mutant-like endosomal abnormalities are produced by reduction of *snn* function in mouse macrophage-like cells (Fig 5). Finally, we have shown that both *hemo* and *snn* display antagonistic genetic relationships with *14-3-3ζ* during endosomal maturation. The binding of Hemotin to 14-3-3ζ offers a direct molecular basis for these interactions (Fig 5). This seems a conserved regulatory mechanism as Stannin peptides have been shown to bind 14-3-3ζ proteins in vertebrates [44] and physical interactions between 14-3-3ζ and the vertebrate PI(3)P enzyme homologues, Mtmr1 phosphatase and class II PI(3)P kinase have been reported by proteomics of 14-3-3ζ pull-down protein extracts from human HeLa cells and mouse neural cells [55,56]. Given 14-3-3ζ multiple functions and near-ubiquitous expression, we surmise that cooperation of 14-3-3ζ with the PI3K68D kinase might be a general cellular mechanism that is modulated by Hemotin/Stannin in specific cell types, although further experiments must test this point.

Interestingly, results relating to the role of Stannin as a promoting factor for organometallic-mediated cytotoxicity may be relevant to our model. Organotins such as TMT are used in industry as plastic stabilisers, but they are acutely toxic, producing cytotoxicity in specific tissues and eventually death of the affected individual [22]. *snn* was originally identified as a cDNA specifically expressed in TMT-sensitive tissues such as hematopoietic organs and immune system lineages [57]. Subsequent studies showed binding of TMT to Stannin [58–60] and of Stannin to 14-3-3ζ [44]. Although the molecular and cellular consequences of such binding and the roles of Stannin in TMT toxicity and under normal physiology have not been fully elucidated, several results might suggest a relationship with PI metabolism. The first cellular symptom of exposure to organometallic compounds is an increase of endolysosomal-like vacuoles [61,62], which resembles both the *hemo* mutant phenotype and the *snn* siRNA phenocopies in mouse macrophage-like cells (S1G and S1H Fig, Fig 5A and 5B). Lithium treatment, which inhibits PI synthesis, has a protective effect on TMT cytotoxicity [62], but addition of exogenous PI(3)P (myophosphatidylinositol) to such TMT+Lithium-treated cells reverts this Lithium protective effect, altogether suggesting that PI promotes TMT-mediated cytotoxicity [62]. Thus, TMT could alter Stannin and cause a toxic excess of 14-3-3ζ-related PI(3)Kinase function, leading to an increase of endocytic PI(3)P labelling, and hence to enlarged endolysosomal compartments and eventual apoptosis in *snn*-expressing tissues.

We have not been able to identify Hemotin-Stannin homologues in diblastic animals or unicellular eukaryotes, so a compatible hypothesis is that Hemotin-Stannin appeared as an adaptation to enhanced levels of endocytic activity in specific phagocytic cells during the evolution of complex body plans. Interestingly, this is also the point when the Sarcolamban family appears in the animal tree [7]. The *sarcolamban*-*sarcolipin* smORF family also encodes peptides regulating a basic cellular process (Calcium homeostasis) fundamental to a specific cell type (muscle cells) [11,63]. smORFs are widespread, having been identified in bacteria [64], yeast [65], and plants [66]. In eukaryotes, translated smORFs have been recently identified in putative long noncoding RNAs [2,3], and interestingly, expression of long noncoding RNAs can be highly tissue-specific ([67], unp. obs.). We have shown that hydrophobic smORF peptides have a propensity to encode transmembrane alpha-helices and to localise to cell membranes and organelles [3]. Finally, it has been proposed that smORFs are a source of evolutionary new peptides [68]. Altogether, it is tempting to speculate that hydrophobic smORFs could provide a source of emerging tissue-specific modulators of organelle-based cellular processes. Regardless of this speculation, the accumulating evidence points to the potential of smORF peptides to fill gaps in our understanding of cell biology and physiology, and its associated diseased states.

## Materials and Methods

### Fly Genetics and Generation of Hemotin A4 Mutant Strain

Fly stocks and crosses were cultured at 25°C. The Oregon-Red line was used as our wild-type strain. The following lines were obtained from the Bloomington Stock Centre: *fray* ^*PZ07551*^, *Df(3)BSC626 (PI3K68D)*, *fru* ^*1*^, *14-3-3ζ* ^*BL12*^, *UAS-Rab7-YFP*, *UAS-2XFYVE-GFP*, *UAS-LAMP1-GFP*, *UAS-PI3K68DIR(GL00159)*.

For ectopic and rescue experiments, we used *Hemese-Gal4*, and *crq-Gal4* lines as described in Sampson et al., 2013 [15].

Fly strains used in this study: *UAS-HA-14-3-3ζ* and *UAS-14-3-3ζIR* [69], *UAS-14-3-3ζIR* (VDRC#48725). *UAS-mtmIR*, *UAS-mtm-Cherry*, *UAS 2XFYVE-Cherry*, *UAS-PI3K68D-Cherry*, *and UAS-PI3K68D-GFP* [21]. *fray* ^*R1*^ [25].

The *P{RS3}fray (CB-0706-3)* and the *P-Bac{WH}fru(f02684)* from DGRC and Exelixis, respectively, were used to generate a 34-Kb deficiency by FRT-mediated recombination [29] depleting the first 5’exon of fru and fray genes and the whole gene locus of *CG7691* and *CG43210* (*hemo*) genes, which was confirmed by Taq-polymerase PCR (Qiagen, Venio, Netherlands) from genomic DNA extractions. In addition, detection of mRNA levels of these genes were conducted by RT-PCR from mRNA extraction using Trizol (Ambion/Life Technologies, Carlsbad, CA) of *hemoA4* larval haemolymph.

### Primary Hemocyte Culture, Live Cell Stainings, Immunocytochemisty and Microscopy

Primary *Drosophila* hemocytes were isolated from postembryonic life stages as indicated in Sampson et al., 2013 [15]. Extraction of free-flowing hemocytes was achieved by bleeding individual specimens using a 25-gauge dissecting needle in a culture medium (80% Schneider’s *Drosophila* medium [Pan-Biotech, Dorset, UK] and 20% fetal bovine serum [Invitrogen/Life Technologies] with no antibiotics).

For measuring vacuolation primary hemocytes from white prepupae (>100 h AEL) were cultured into a glass-bottom dish containing collagen extracellular matrix (MatTek Corp., MA, US) and stained with anti-beta tubulin and phalloidin (Invitrogen/Life Technologies) following the procedures described in Sampson et al., 2013. Similar procedures were followed to identify lysosomes using lysotracker (Invitrogen/Life Technologies) or by detecting the LAMP1-GFP marker.

Detection of GFP and Cherry–2XFYVE, Hrs, Cherry-PI3K68D, Cherry-Mtm, HA-14-3-3ζ, and hemo-ORF1-GFP was achieved by culturing primary hemocytes from wandering late third instar larvae and white prepupa stages in glass multisport microscope slides (Hendley-Essex, Essex, UK) and immunostaining was performed as described in Sampson et al., 2013.

Antibodies used in this study are: mouse anti-GFP (Roche) 1:500; rabbit anti-cherry (Invitrogen) 1:5000; mouse anti-HA (Roche) 1:500; anti-Hrs [70], mouse anti-FLAG M2 (SIGMA) 1:1000. Actin cytoskeleton was labelled with fluorophore-conjugated phalloidin (Molecular-Probes. Invitrogen) 1:50.

For measuring lysosomal aggregation, live primary hemocytes were stained with 50 nM Red-lysotracker (Invitrogen) in culture medium for 15 min and then washed several times and finally replaced with normal culture medium for live imaging for 20–30 min.

For embryonic hemocyte *in vivo* imaging, adult flies were allowed to lay eggs on apple juice agar plate. Embryos collected at stage 15, dechorionated and mounted on double-sided tape stuck to a standard glass slide. Voltalef oil was applied to the embryos to prevent dehydration, and a coverslip fixed with nail polish applied on top. For pupal hemocyte *in vivo* imaging, pupae at stage P6 (50 h APF) were mounted ventral side down on double-sided tape applied to a glass slide and dissected to remove a window in the pupal case over the thorax as described in Sampson et al., 2013. After application of 10S Voltalef Oil on the ROI, a ring of petroleum jelly was made around the samples, a coverslip was rested on this ring, and then it was pressed down on the sample.

Confocal laser microscopy was used to image fluorescence in live and fixed cells. An inverted Zeiss Axiovert 200M series microscope with a LSM-510 confocal laser attachment was used. Images were captured using a Hamamatsu ORCA-ER C4742-95 camera. Most cells were observed at 63x using the Zeiss Apochromat 63x oil objective NA = 1.4, but in some cases 100x magnification was required, using a Zeiss Epiplan—NeoFluar 100x oil objective with NA = 1.3. Fluorescent imaging utilised the automated prior stage for Z-stacking throughout the cells imaged. Live cells were imaged at 0.5–1 μm path length between each imaging slice. Fixed cells were also imaged within the same range of path length for nondetailed images, whilst detailed images, particularly for 3-D reconstruction, were imaged at a path length of 0.2–03 μm between each imaging slice. LSM image browser and imageJ v.1.46 were used to analyse acquired images and Photoshop (Adobe) was used for editing.

### Bacterial Phagocytosis Assay in Primary Hemocytes

Phagocytosis assays were performed on wild-type and *hemo* ^*A4*^ mutant live primary hemocytes expressing FYVE-GFP endocytic marker supported in *ex vivo* cell culture without ECM coating and using HEPES+HBSS pH 7.4. The phagocytic stimulation used was in the form of pHrodo pH-sensitive conjugated heat-killed bacterial particles. *E*. *coli* (Gram -) bio-particles were used (Life Technologies).

A 1:1 dilution of pHrodo-conjugated bacterial particles (0.5 mg/mL) with HEPES+HBSS pH 7.4 buffer was sonicated for 5 min to break apart large conglomerates. Further dilution was carried out by using 16 μl (1:1 dilution) into a final volume of 200 μl HEPES+HBSS pH 7.4 which was added to the *ex vivo* hemocyte culture. Time-lapse Imaging was conducted using a SP8 Leica confocal microscope with a short Z-stack throughout the cell at 0.5 μm path length between each image slice. Bright Field Phase, 488 and 543 nm laser illuminations were used to observe the cell outline and the fluorescent FYVE and pHrodo particles. Live cells were imaged every 90 to 120 seconds with a Z-stack for a period lasting for 90 min.

The integrated pHrodo intensity over time was measured for each particle from the moment the particles docked into the cell membrane and for each time point, using an identical ROI of 0.9 μm^2^ for every series on single confocal z-stack plane across on the middle of the pHrodo particle. To quantify FYVE prevalence, the number of frames for which individual pHrodo particles were surrounded by FYVE, using a single z-stack plane across on the middle of the pHrodo particle, were counted and converted into time according to the frame rate of each time lapse.

### Viability Assays

For viability assays, virgin male flies were collected from each genotype and kept at 18°C until sufficient numbers were reached (maximum 2 d, at least 50 flies per genotype or condition). Flies (2-d-old) were then kept at a density of 10–25 flies/tube, reared at 25°C and scored daily for number of surviving flies.

To assess the viability of flies in antibiotic media, 100 μL of Penicillin-Streptomycin solution (10,000 units penicillin and 10 mg streptomycin/mL, *Sigma-Aldrich*) was added to the surface of standard corn-meal food media vials (containing approximately 10ml of media), and left over night at 25°C, until fully absorbed.

To assess the effect of bacterial infection on viability, nonpathogenic *Escherichia coli* (DH5α), or pathogenic *Micrococcus luteus* (gram+) and *Enterobacter cloacae* (gram−) bacterial strains were used. Flies were infected by wounding at the top of the abdominal segment, under the haltere, using a tungsten dissection needle dipped in bacterial culture grown to a cell density of (OD600 = 0.8).

### In Vivo *mCherry E*. *coli* Uptake Assay

To quantify the number of *mCherry E*. *coli* bacteria (*K12 E*. *coli* expressing *PDSpRSETD-cherry* plasmid from Dr. Stephan Mesnage) in hemocytes *in vivo*, *He-Gal4; UAS-FYVE-GFP* or *he-Gal4; UAS-FYVE-GFP*, *hemo* ^*A4*^ flies were injected with 0.2 μL of bacterial culture grown to OD600 = 0.05, using a glass capillary microinjection needle. Flies were then dissected as described in Magny et. al 2013 [7] to expose the dorsal abdominal cuticle in order to image the hemocytes associated to the dorsal vessel, as described in Horn et al. 2014 [71]. Flies were dissected after 20 or 120 min postinjection, in PBS, and the cuticles fixed in 4% PFA in PBS for 20 min. The preparations were then washed in, PBTX, incubated 30 min in PBS with phalloidin-Cy5 (*Sigma-Aldrich*) (1:10), washed in PBS, and mounted in Vectashield *(vector*). Hemocytes were imaged with a Zeiss laser scanning microscope LSM 5.10 on a Zeiss Axioskop 2 stage, with a 40X Achroplan objective, and bacterial cells within hemocytes, as determined with the phalloidin and FYVE counter-stains, were counted in Z-stack reconstructions.

### Endogenous Bacterial Content Assay

To quantify endogenous bacterial contents, we followed the method described in Khalil et al. 2015 [72]. Briefly, *hemo* ^*A4*^ and wild-type *Oregon Red* L3 larvae were collected, washed in PBTX and PBS, and reared together in the same vial at a density of 20 flies per vial, in order to minimize the effect of external bacteria present in the media. Wild type and mutant flies were collected at either 10 d or 30 d after eclosion, and homogenised individually in 100 μL of autoclaved and sterile filtered PBS. The homogenates were diluted serially, and plated in antibiotic-free LB-agar plates. The plates were incubated for 48 h at 29°C, and the bacterial colonies from each homogenate spot counted.

### Generation of DNA Constructs

The full-length *hemo* cDNA was obtained by RT-PCR (RACE) (PCR from overlapping EST (Flybase) using specific primers and cloned in TOPO vector (Invitrogen). *pUAST-hemo* full length vector was constructed by inserting flanking restriction sites to the 5’ and 3‘ end of the *hemo* cDNA respectively by PCR amplification. *hemo* cDNA was inserted in the *pUASt (AttB)* vector using these restriction sites. A similar strategy was used to clone ORF1 and ORF2 into the *pUASt (AttB)* vector. Generation of the *pUASt-hemo-ORF* carboxyl-tagged with GFP was carried out by generating a single PstI restriction site at the end of the ORF removing the stop codon in the *hemo* cDNA in TOPO by PCR and subsequently inserting a PstI flanking GFP cds in frame lacking its kozak and methionine sequences. Then the hemo-GFP was cloned in the *pUASt*-vector. The *pUASt (AttB)-hemo-GFP* was constructed for site-directed transgenesis by removing the *hemo-GFP* from *pUASt* and cloned in the *pUASp(ATTB)* vector (DGRC). *The pUASt-ORF2* carboxyl terminal GFP tagged construct was made by amplification of ORF2 sequence and a short 5’UTR according to the short *hemo* cDNA described in flybase by PCR. The PCR product was cloned in pENTR vector (Invitrogen). LR recombination (Invitrogen) was used to clone the ORF2 into the *pUASt-ctGFP* vector (Murphy collection, Carnegie Institute).

The *hemo* frameshift construct was engineered by introducing a double nucleotide insertion in each ORF (in ORF1 a CG insertion three codons after the ATG and in ORF2 a GC insertion just after the ATG) by site directed mutagenesis (Stratagene, CA, USA) using the *hemo* full length cDNA in TOPO as a template. Subsequently, the *hemo* frameshift cDNA was cloned in *pUASt (ATTB)* vector.

A *CG7691* Genomic Fragment spanning 11.8 Kb was constructed by amplifying independently four sequential genomic fragments by Long Expand Range PCR (Roche, Basel, Switzerland) each containing the EcoRI-Kpn1, KpnI-NotI, NotI-XbaI and XbaI-AscI restriction sites respectively and then cloned in TOPO vector (Invitrogen). Each fragment was sequentially cloned in the Casper 5 vector using the restriction sites. Proof of the presence of the wild-type the *CG7691* gene was obtained by sequencing. *CG7691* mRNA expression from the construct was detected in adult transgenic flies over an *hemo* ^*A4*^ deletion background by RT-PCR as described in [7].

To generate an amino terminal tagged version of 14-3-3ζ the ORF and 3’UTR from *RH61958* cDNA (DGRC) were amplified by PCR and subsequently the PCR product was cloned in the *pENTR* vector (Invitrogen). The *pUASt-Ntmyc-14-3-3ζ* construct was generated by LR recombination into the acceptor vector (Murphy’s collection; Carnegie Institute).

A *snn* ORF clone in an entry vector (GeneCopoeia, MD, US) was inserted in the *pUASp* vector (Murphy’s collection, Carnegie Institute) by LR recombination (Invitrogen). Similarly, a *pUASt*- amino-terminal GFP- or FLAG-tagged SNN constructs were generated using the former *snn* ORF entry vector and destination vectors (Murphy’s collection, Carnegie Institute).

To generate the *hemo-*RNAi construct used the method described by Kondo et al. (2006) [73]. Briefly, we used the following primers (*hemo* RNAi Fw GTTCCACAGAGATATCGTCT *hemo* RNAi Rv ACCACGAAGCTAACGCACAGC) to amplify a 354 bp DNA fragment from genomic DNA, corresponding to a region of the *hemo* locus with no homology to other genomic regions. This fragment was then cloned into the *pRISE* vector and used to generate transgenic flies by Bestgene.

### *Drosophila* S2 and Kc167 and Mouse RAW264.7 Cell Culture

S2 cell culture, transfections, and immunocytochemistry were performed as described in [7]. Kc167 cells (250X10^5^) were cultured in a 6-well plate dish in M3 insect medium (Sigma, MO, USA) containing 10% FBS and 1% pellicillin/streptomycin (Sigma). After 24 h, *pUASt*- and *Act5-Gal4*-constructs were transfected using FuGene (Roche). 3 d later, cells were pelleted for immunoprecipitation.

Mouse RAW264.7 cells were cultured in RPMi-1640 with L-glutamine (Sigma) supplemented with 10% FBS and 1% penicillin-streptomycin (Sigma) at 37°C and 5% CO_2_ conditions. 300 x 10^5^ cells/well were grown in a 6-well plate (for lysotracker they were placed on acid-treated coverslips). siRNA treatments were performed as described by Ulvila et al., 2011 [43]. A mixture of two 5’ FITC labelled siRNAs were generated for *snn*, one targeting the ORF (sense sequence-GGCCAUGUGUGGAAAGAAAUU) and the other the 3’UTR(sense-sequence-GGGAGGAGCUGUAGGGAAGUU), and a control sample of nontargeting siRNAs pool manufactured by Dharmacon (GE Healthcare, NJ, US) were transfected using RNAmax reagent (Invitrogen) into cells at 24h and 48h after seeding the cells. After 24 h, cells were pelleted for Trizol (Ambion) mRNA extraction. Retrotranscription was conducted using random and polydT primers using RNA isolation kit (Promega, CA, US). *snn* and *elongation translation factor 4* primers were used for RT-PCR. For quantification of lysosomes with Lysotracker, the media was removed and cells were washed with PBS1x. Afterwards, 50nM Lysotracker in culture media was added and left for 15 min. Cells were washed with culture media and mounted for acquisition of live cell imaging (30min) using confocal microscopy with a Zeiss LSM 510 NLO AXIOSkop microscope. Three independent siRNA treatment repeats were performed in our analysis.

### Immunoprecititation and Western Blot

Late third instar larvae (80) and pelleted Kc167 cells were homogenized in lysis buffer (50 mM Tris pH 7.5;150 mM NaCl;1 mM EDTA;1 mM EGTA; 2.5 mM pyrophosphate; 1 mM Na_3_VO_4_; 1 mM glycerol phosphate) for 1 h (4°C). Cellular debris was spun at 3,000 rpm for 15 min at 4°C. Supernatant was again spun at 9,500 rpm for 45 min at 4°C. Supernatant was added to equilibrated GFP-beads (Chromo Tek, NY, US) and left rotating overnight at 4°C. Beads were washed several times and then boiled in Laemmi Loading Buffer (Biorad, CA, US). Beads were loaded onto 8% or 12% polyacrylamide gel and proteins were separated by SDS-PAGE (Biorad). Specific proteins were detected by Western Blot using a semidry blotting or tetra cell (Biorad). Antibodies used were: mouse anti-GFP 1:3000 (Roche); anti-HA 1:5000 (Roche); anti-Myc 1:1000 (Upstate).

### Statistical Analyses of Cell-Based Assays

To measure the size of the vacuoles of *in vivo* embryonic and pupal thoracic hemocytes, we calculated the OAI, which is the accumulative area of intracellular vacuoles divided by the cellular area (>30 cells for most of the genotypes randomly selected as described above) in hemocytes in the wild-type and *hemo* ^*A4*^ mutant. Z-stack images were processed in the ImageJ software, and the outlines of the vacuole and cell were highlighted using the draw tool and the areas were measured. Student *t* test was used for statistical analysis.

For measuring vacuolation in *ex vivo* hemocytes, we determined the OVI, which corresponds to the addition of the volume of intracellular vacuoles (voids that disrupt the tubulin cytoskeleton)/total cytoplasmic volume of the cell. The Z-stack images were loaded onto the ImageJ software. To calculate the total cytoplasmic volume, we used the oblate ellipsoid volume formula: 4/3πab^2^ (a = radius, b = height). The radius of the cytoplasm was measured at widest Z-slice of the cell and the height at the highest point at the orthogonal projection. The average wild-type cytoplasmic volume of *ex vivo* hemocytes (>30 cells for most of samples were selected at random across >3 separate imaging foci and 3 experimental repeats) was 311.06 μm^3^, which diverged only slightly from A4 mutants (375.57 μm^3^), therefore the wild-type cytoplasmic volume was used for our analysis. To calculate the volume of intracellular vacuoles, we used the sphere volume formula: = 4/3πr^3^, the radius was calculated by measuring the diameter of the widest point of the vacuole, only the vacuoles with a radius equal to or larger than 1.5 μm were considered for OVI analysis, with cells lacking vacuoles of at least 1.5 μm giving an OVI of 0.

To measure early endosome cellular compartments (FYVE-positive) in *ex vivo* hemocytes, we calculated the OAI, meaning the addition of FYVE particle area/total cytoplasmic cell area (volume inside of cortical actin ring). ImageJ software was used to calculate the OAI. A maximum projection of the Z-stacks was made and both FYVE and Phalloidin channels were separated. FYVE image was transformed into a binary image and particle analyses (size pixel μ^2^: 20-infinity and circularity: 0–1) were used to calculate the area of particles. Addition of FYVE particle areas was done in Excel. Measurement of the cytoplasmic area was performed manually by using the free hand selection tool. OAI of endosomes of 20–60 cells selected at random as described above were calculated per genotype. In addition, we measured FYVE diameter ratio consisting of the average of three FYVE vesicle diameters per cell (randomly selected 20–60 cells from >3 imaging foci and 3 experimental repeats per genotype) divided by the averaged FYVE diameter of the *He-Gal4 UAS-hemo* full-length transcript in an A4 mutant background to diminish the genetic background effects possibly caused by the *Gal4*, *hemo* ^*A4*^ and *UAS*-docking chromosomes.

Lysosome aggregates in *ex vivo* hemocytes were measured by calculating the area of lysosome particles as described above (visualized with lysotracker)/number of lysosomal particles. For each genotype >30 cells were selected randomly as described above and were measured.

We calculated the OAI as described above as a measure of the lysosome occupied area in the siRNAs experiments in mouse RAW264.7 cells. 80–110 cells were measured per sample in each of the three repeats.

Statistical analysis was performed with one-way ANOVA to assess whether the means of the groups were significantly different (*p* < 0.0001), and a post hoc Bonferroni’s test was carried out to compare multiple groups with *p* < 0.05 considered as significant using the Prism suite (Graph Pad).

As a measure of the colocalisation of endocytic markers and hemotin-GFP peptides, we used the Pearson’s correlation coefficient (ImageJ WCIF colocalisation plugins) that evaluates the amount of signal intensity from one channel that occurs in the same location in the other colour channel. Pearson’s coefficients range between −1 to 1, with values closer to 1 indicating reliable colocalisation. Pearson’s coefficients of hemocyte cells (*n* = 17–32 per genotype) were analysed with two-tailed Mann-Whitney test (*p* < 0.0001).

### Search for Sequence and Structure Homologues Using Bioinformatics

The Phyre2 online search engine was used to find structural homologues using the Hemo-ORF peptide sequence as an input. To find Hemotin sequence homologues, we used the pipeline described in Magny et al., 2013 [7] that is based on the identification of closest homologues, followed by expanded searches using consensus sequences weighted by the phylogeny from the alignment of such close homologues. In addition, we introduced an extra corroboration step of reciprocal best Blast hit in doubtful cases.

Alignments and trees of peptide sequences were generated using MAFFT and Clustal programs, with MAFFT parameters set at 5 iterations and global pair alignment. The alignments were visualized with Jalview.

To evaluate the ability of hemo peptides to adopt the tertiary peptide structure of human Stannin, we threaded the sequence of insect Hemotin peptides (*D*. *melanogaster* and *Microplitis demolitor*) and the basal vertebrate Stannin peptide (hagfish) onto the human Stannin peptide structure (1zzA; RCSB protein data bank) using the Phyre2 server [35] on one-to-one threading mode. These preliminary structures were then refined, and Global distances test values were obtained with respect to the original human 1zzA Snn structure using the KobaMIN server [38].

## Supporting Information

S1 Data Excel spreadsheet containing the numerical data and statistical analysis, shown in separate sheets for: Fig 1O. OVI vacuolation in *hemo* loss of function (LOF) and rescues. Fig 2D. FYVE OAI in *hemo* LOF and rescues. Fig 2E. Pearson’s correlation coefficients between FYVE and Lysotracker in wild-type and hemo^A4^ mutants. Fig 2F. Pearson’s correlation coefficients of Hemo-GFP with FYVE and Lysotracker. Fig 3D. Measurements of pHrodo intensity in wild-type and hemo^A4^ mutants.Fig 3E. Prevalence of FYVE signal in pHrodo particles. Fig 3J. Average of bacteria expressing mCherry inside hemocytes. Fig 3L. Average of endogenous bacteria in wild-type and *hemo^A4^* mutants. Fig 3M. Percentage of mortality of wild-type and *hemo^A4^* mutants in different conditions. Fig 5C. Lysotracker OAI values of nontransfected, control and *snn*-siRNAs. Fig 5G. OVI vacuolation values for *hemo*, *snn*, and *14-3-3ζ* interaction. Fig 5H. FYVE OAI values used for hemotin, *snn*, and *14-3-3ζ* interaction. Fig 6J. FYVE OAI values for *hemo*, *14-3-3ζ*, *mtm*, and *PI3K68D* interactions. S1K Fig. Aggregation of Lysotracker particles values of *hemo* LOF and rescues. S2B and S2C Fig. Pearson’s correlation coefficients between Rab7 and Lysotracker markers in wild-type and *hemo^A4^* mutants. S2D and S2E Fig. Pearson’s correlation coefficients between Rab7 and FYVE in wild-type and *hemo^A4^* mutants. S3A Fig. Average number of pHRODO particles phagocytised per hemocyte after 20 and 40 minutes postinjection in wild-type and *hemo^A4^* mutants. S3C and S3D Fig. Effect of bacterial infection on the life-span of wild-type flies and *hemo^A4^* mutants. S5B Fig. Quantification of *snn* expression in Raw 264.7 cells, with and without *si-snn* treatment. S6 Fig. Ratio of average diameter of 3 FYVE vesicles per cell divided by the average *hemoFL* rescue value.
(XLSX)

S1 Fig *hemotin* sequence, predicted secondary structure, and phenotypic analysis. (A) cDNA sequence of the *hemo* transcript. The amino acid sequence of the peptide encoded by each ORF is shown underneath of DNA sequence and whose start codons are highlighted in bold. *hemo*-ORF encodes an 88aa peptide (navy blue), and ORF2 encodes a 59aa peptide (light blue). The fragment underlined in the *hemo*-ORF sequence corresponds to the sequence detected by proteomics in membrane fractions of *Drosophila* S2 cell extracts (Brunner et al., 2007). (B) Bioinformatic prediction of transmembrane helices in Hemo-ORF (top) and human Stannin (bottom) by Hidden Markov Model for Topology Prediction (HMMTOP) program [37]. Note that each peptide contains a single transmembrane helix (blue box). (C) RT-PCRs, using mRNA extracted from hemocytes bled from wandering larvae. The hemocyte-specific gene *Hemese (He)*, and *rp49* control are expressed at similar levels in wild-type and *hemo* ^*A4*^ mutant hemocytes. *hemo* is expressed in wild-type, at comparable levels to *He*, but is completely absent in *hemo* ^*A4*^ mutant hemocytes. (D–E) Embryonic hemocytes from the dorsal side of stage 15 embryos expressing *crq-Gal4;UAS-GFP* in wild-type (D) and *hemo* ^*A4*^ mutants (E). In *hemo* ^A4^ mutants, embryonic hemocytes appear to have enlarged vacuoles within the cytoplasm (arrowheads). Scale bar (10 μm). (F–F”) Expression of Hemo-GFP (green) (F”) and the membrane marker mCherry-CD8 (red)(F’) UAS-constructs driven by *Act5-Gal4* in Kc 167 cells (green). The Hemo-GFP peptides colocalise with CD8 proteins in the membrane of intracellular compartments (arrow) (F). Scale bar (5 μm). (G–J) Detection of acidic pH-sensitive Lysotracker in *ex vivo* prepupal hemocytes. Scale bar (5 μm). *hemo* ^A4^ mutants (H) display larger Lysotracker-positive organelles (arrowheads) than wild-type (G). The enlarged *hemo* ^A4^ lysosomal vesicles are fully rescued by expression of *hemo* full length transcript (*UAS-hemoFL*) (I) or by the expression of the *hemo*-ORF mini-gene (*UAS-hemo-ORF*) (J). These UAS constructs were driven by *He-Gal4*. (K) Quantification of lysosomal aggregation showing that *hemo* ^*A4*^ mutant hemocytes contain significantly more Lysotracker aggregation per cell than wild-type (see S1 Data), and that both *hemoFL* and *hemo-ORF* rescue this phenotype. A one-way ANOVA test show that the mean of these samples are significantly different [F(3,103) = 37.93, *p* < 0.000] and a post hoc Bonferroni multiple comparison test between genotypes showed that hemo^A4^ was significantly different to wild-type, whereas *UAS-hemoFL-hemo* ^*A4*^ and *UAS-hemoORF-hemo* ^*A4*^ were not (*n* ≥ 22, *p* < 0.05). Error bars represent SEM. Supplemental data are shown in S1 Data.
(TIF)

S2 Fig The Hemotin peptide is required for proper endosomal maturation in hemocytes. (A–A”) Distribution of the LAMP1-GFP lysosomal marker (green) (A’) and the cytoskeletal protein beta-tubulin (red) (A”) in wild-type *ex vivo* hemocytes. LAMP1-GFP positive compartments are scattered throughout the cell without large disruptions of the tubulin cytoskeleton. Compare with *hemo* ^*A4*^ mutant hemocytes in Fig 2A–2A”. Scale bar (5 μm). (B–B”) Localisation of the late endosomal marker Rab7-YFP (green) (B, B’) and acidic pH-sensitive Lysotracker (red) (B, B”) in a wild-type *ex vivo* hemocyte, showing a considerable overlap between these two markers as expected (11). Median of the colocalisation Pearson’s correlation coefficients (P.C) obtained with a two-tailed Mann-Whitney test of 17 cells. Scale bar (10 μm). (C–C”) Large Rab7-YFP (C, C’) and Lysotracker (C, C”) intracellular compartments (arrow heads) in *hemo* ^*A4*^ mutant *ex vivo* hemocytes, labelled as in (B). Median of Pearson’s coefficient obtained as in B, showing significantly larger colocalisation between these two compartments than wild-type (*n* = 17, *p* < 0.0001). Scale bar (10 μm). (D–D”) Expression of the FYVE early endosomal (red) (D”) and the Rab7 late endosomal (green) (D’) markers in *ex vivo* hemocytes shows that there is an overlap between FYVE and Rab7 compartment. Median of the Pearson’s correlation coefficients calculated with a two-tailed Mann-Whitney test (*n* = 21). (E–E”) Enlarged intracellular vesicles (arrowheads) expressing an overlap of Rab7-YFP (E’) and FYVE-Cherry (E”), markers that characterise late endosomes, are observed in *hemo* ^*A4*^ mutant hemocytes. (E) Merged image. The median of Pearson’s correlations coefficients is significantly larger than wild-type obtained with a two-tailed Mann-Whitney test (*n* = 31, *p* < 0.0001). Scale bar (10 μm). (F–G) Rab11-GFP positive compartments have similar sizes in wild-type hemocytes (F, F’) and in *hemo* ^*A4*^ mutant hemocytes (G, G’). Actin cytoskeleton revealed by phalloidin staining (red). Scale bar (5 μm). (H) Hemo-GFP peptides (H”) colocalise with early endosomal Hrs protein (H’) in *ex vivo* hemocytes. (H”) Merged image. Scale bar (5 μm). Supplemental data are shown in S1 Data.
(TIF)

S3 Fig Phagocytic rate of *hemo* ^*A4*^ and wild-type hemocytes and effect of bacterial infection on the life span of *hemo* ^*A4*^ and wild-type flies. (A) *hemo* ^*A4*^ mutant (red) and wild-type hemocytes (blue) internalise similar numbers of pHrodo particles after 20 or 40 min, suggesting that phagocytosis occurs at a similar rate in wild-type and mutant hemocytes. Error bars represent SEM. (B) Table showing the average median life span in days (+/–standard deviation) of flies after infection with different bacterial strains (flies were infected by wounding with a needle dipped in bacterial culture grown to OD600 = 0.8). *Fob1* is a mutant blocking digestion of phagocytised bacteria [33], and *Imd* mutants abrogate the humoral response (production of antibacterial peptides) upon infection by gram− bacteria. (C) Graphic representation of the reduced viability in *hemo* ^*A4*^ mutants compared to wild-type. The median life span for each genotype is plotted relative to the average median life span of wild-type flies. Error bars represent SEM. (D) Graphic representation of the effect of bacterial infection on life span in *hemo* ^*A4*^ mutants (red) and wild-type flies (blue). The median life span for each condition is plotted relative to the average median life span of noninfected flies for each genotype. The life span of *hemo* ^*A4*^ mutant flies is dramatically reduced by infection with normally nonpathogenic *E*. *coli*, but not by *M*. *luteus* or *E*. *cloacae*, two pathogenic strains that bypass the cellular defence of hemocytes but are fully engaged by the humoral immunity of antibacterial peptides [13]. In wild-type flies, the three bacterial strains lead to a milder reduction in life span. For each condition, two to five different replicates were analysed, for a total of 50–125 flies. Error bars represent SEM.
(TIF)

S4 Fig Hemotin is a member of the conserved Stannin family. (A) Sequence alignment of all the identified insect Hemotin and vertebrate Stannin peptide sequences (see S4 File), labelled as in Fig 4A). The most conserved region of these sequences is the N-terminal hydrophobic region (light blue) corresponding to the transmembrane α-helix domain. Previously annotated Stannin sequences are prefixed by (snn_). (B) Phylogenetic guide tree showing average distances between the insect Hemotin and vertebrate Stannin peptides. The length of the branching lines is proportional to the distance between sequences. The Hemotin and Stannin sequences cluster with their respective counterparts from related species in their own taxa (Hymenopteran and Dipteran insects, and Vertebrates). The location of the sequences in the tree follows the phylogenetic relationships among these animals, i.e., within each taxon cluster, the sequences from more ancestral species locate to more basal positions in the tree, except occasional and highly-divergent sequences. The tree also correctly shows the 88aa mitochondrial ribosomal protein S21 (mRpS21) (which is conserved across these taxa) and an unrelated outgroup. Previously annotated Stannin sequences are prefixed by (snn_). (C) Amino acid sequence similarity scores (obtained with the Clustal program) between human Stannin and sequences whose structural compatibility has been examined (Fig 4C). Maximum and minimum scores across the three taxa examined (dipteran, hymenopterans, and vertebrates), plus data for the Sarcolamban family of smORF peptides [7] are shown for comparison. Sequence similarity between vertebrate Snn peptides and insect Hemo peptides is within the range of minimal scores within vertebrate Snn peptides and within the sarcolamban (Scl)-Phospholamban (Pln)-sarcolipin (Sln)- family. Structural and functional homology between insect Hemotin and vertebrate Stannin peptides (green rows) can be observed with sequence similarity scores below their intertaxa maxima (blue rows). Full scores of sequences used in Fig 4B are in S3 File. Supplemental data are shown in S3 File and S4 File.
(TIF)

S5 Fig Quantification of siRNA *snn* knockdown, myc-14-3-3ζ and Hemo-GFP pull-down input, and membrane localisation of Snn-GFP. (A) Agarose gel showing RT-PCR products, corresponding to the *snn* or *etf4* (control) transcripts, from mRNA extracts of mouse macrophage-like RAW264.7 cells treated with *snn*-specific siRNA, or with scrambled siRNAs control, compared to nontransfected cells. *snn* siRNA effectively reduces the levels of *snn* transcript compared to non-transfected cells, whereas control siRNA have a negligible effect on *snn* expression. *etf4* shows comparable levels of expression in all conditions. (B) Quantification of the intensity levels of *snn*-specific RT-PCR band relative to *etf4*, from the gel described in (A). Average measurements were obtained from three independent experiments. Error bars represent SEM. (C) Western Blot denoting the Hemo-GFP and GFP proteins retained by GFP beads in the Pull down experiment shown in Fig 5F. (D-D”) Expression of Snn-GFP peptides and membrane CD8-mCherry proteins using the *Act5-Gal4* driver in Kc167 cells. Snn-GFP positive intracellular compartments colocalize with the CD8-mCherry membrane marker (arrows). Note that there exists other intracellular membranous compartments not labelled by Snn-GFP peptides. Supplemental data are shown in S1 Data.
(TIF)

S6 Fig Diameter ratio of FYVE vesicles in primary hemocytes. The diameter ratio of FYVE vesicles was calculated as the average diameter of three representative vesicles per cell divided by the average diameter of the *He-Gal4;UAS-hemotin FL; hemo* ^*A4*^ rescue, in order to diminish any effect of genetic background of *hemo* ^*A4*^, *He-Gal4* and *UAS-* chromosomes in the measurements (see S1 Data). FYVE was labelled using either *UAS-FYVE-GFP* or *UAS-FYVE-RFP*. *hemo* ^*A4*^ mutant vesicles are twice the size of wild-type vesicles. The enlarged *hemo* ^*A4*^ mutant FYVE diameter phenotype is rescued by expression of *hemo* full length transcript (*UAS-hemoFL*), the *hemo-ORF* mini-gene *(UAS-hemo-ORF*) and *snn* (*UAS-snn*). Knocking down the expression of *14-3-3ζ* (*14-3-3ζ-RNAi*) reduces the *hemo* ^*A4*^ enlarged FYVE vesicle-diameter even further than the *hemo* full-length rescue. Conversely, over-expression of *14-3-3ζ* (*UAS-14-3-3 ζ*) produces larger FYVE vesicles, similar to those in *hemo* ^*A4*^ mutants. Over-expression of *hemo* full length transcript rescues the large FYVE vesicles induced by excessive *14-3-3ζ*. Removing a single copy of the *PI3k68D* gene (*Df(3)PI3K68D*/+) reduces the size of *hemo* ^*A4*^ FYVE vesicles. On the contrary, over-expression of *PI3K68D* (*UAS-PI3K68D*) produces larger FYVE vesicles. Over-expression of *hemo* full length transcript rescues the enlarged FYVE vesicles produced by over-expression of *PI3K68D*. Loss of function of *mtm* (*mtm-RNAi*) causes an enlargement of FYVE vesicles. Over-expression of *mtm* (*UAS-mtm*) rescues the enlarged *hemo* ^*A4*^ mutant FYVE vesicle size. Knocking down the expression of *14-3-3ζ* (*14-3-3ζ-RNAi*) suppresses the *mtm* loss of function and *PI3K68* gain of function phenotypes. Finally, reduction of *PI3k68D* function (*UAS-PI3k68D-RNAi*) rescues the enlarged FYVE vesicles produced by over-expression of *14-3-3ζ*. One-way ANOVA test showed that means of samples are significantly different [F(15,700) = 64.62, p<0.0001]. A post hoc Newmann-Kleuss Multiple comparison test indicate the significance between genotypes shown in S1 Data (n≥19*, p<0.05). * The number of cells in *UAS-mtm;hemo* ^*A4*^ was lower due to lack of cells having vesicles with the minimum size required for this analysis (see Materials and Methods). Error bars represent S.E.M. Supplemental data are shown in S1 Data.
(TIF)

S1 File Supplementary gene expression analysis and *in vivo* hemocyte pull-down. (DOCX)

S2 File Hemo-GFP and ORF2-GFP expression in *Drosophila* S2 cells and phenotypic analysis of segmental nerves in *hemo* ^*A4*^ *and fray* allelic combinations. (DOCX)

S3 File Percent identity matrix created by Clustal2.1. (DOCX)

S4 File FASTA file with all Hemotin and Stannin amino acid sequences used in this study with their respective accession numbers. (TXT)

S1 Video Time-lapse of a wild-type hemocyte expressing FYVE-GFP in ex-vivo culture supplemented with *E*. *coli* pHrodo bacterial particles. (AVI)

S2 Video Time-lapse of a *hemo* ^*A4*^ hemocyte expressing FYVE-GFP in ex-vivo culture supplemented with *E*. *coli* pHrodo bacterial particles. (AVI)

## Abbreviations

crq: croquemort
DAPI: 2-(4-Amidinophenyl)-6-indolecarbamidine dihydrochloride
FISH: fluorescent in situ hybridisation
FITC: Fluorescein isothiocyanate
FYVE: PI(3)P binding zinc finger domain, early endosomal marker, named after being found in Fab1, YOTP, Vac1, EEA1
GDT: High-Accuracy Global Distance Test
GF: genomic fragment
GFP: green fluorescent protein
*He*: *Hemese*
*hemo*: *hemotin*
hemoFL: *hemo* full-length transcript construct
hemoFS: hemo full length transcript construct containing frameshifts in hemo ORF and ORF2
LOF: loss of function
Mtm: Myotubularin
OAI: occupied area index
ORF: Open Reading Frame
ORF2: hemo ORF2 mini-gene construct
OVI: occupied volume index
PI: phosphatidylinositol
PI(3)P: phosphatidylinositol-3-phosphate
PI3K68D: Phosphatidylinositol 3 kinase 68D
ROI: region of interest
RPKM: Reads Per Kilobase of transcript per Million mapped reads
RT-PCR: Reverse transcription polymerase chain reaction
SEM: standard error of the mean
siRNA: small interfering RNA
smORF: small Open Reading Frame
Snn: Stannin
TMT: trimethyltin
UTR: untranslated region
UAS: upstream activating sequence
Vps34: Phosphatidylinositol 3 kinase Vps34
