# Supplementary material for: Hemotin, a Regulator of Phagocytosis Encoded by a Small ORF and Conserved across Metazoans
Source: PLoS Biol. 2016 Mar 25;14(3):e1002395. doi: 10.1371/journal.pbio.1002395 (PMC4807881; doi:10.1371/journal.pbio.1002395)

**SUPPLEMENTARY FILE 1**

Supplementary gene expression analyses and *in-vivo* hemocyte pull-down.

(A) Expression profile of *hemotin (hemo*) during development, assessed by RT-PCR with mRNA extracts from 0-24h embryonic stages (E), first, second and third (1, 2, 3) larval instars, white to undifferentiated pupae (P), and adults (A). The expression of *hemotin* is regulated temporally, being low, although detectable, in embryonic and larval stages, and increasing in pupal and adult stages. The expression of the *rp49* control remains constant throughout development. This is in agreement with the modEncode RNAseq data (<http://flybase.org/reports/FBgn0262823.html>; as accessed in February 2016)

(B) mRNA expression of *CG7691, fray* and *fruitless* in wild-type (Or-R) and *hemo^A4^* mutant hemocytes. *CG7691* and a short *fray* isoform (containing the full Fray coding sequence) are expressed in wild-type hemocytes, but *fruitless* is not. In *hemo^A4^* mutant hemocytes, the expression of *CG7691* is lost but the expression of the short *fray* isoform remains.

(C) Transgenic flies carrying the *CG7691* genomic fragment rescue construct in a *hemo^A4^* mutant background (2: *CG7691GF;hemo^A4^)* show similar levels of expression of *CG7691* as wild-type (1: Or-R). The smaller band on the *CG7691GF;hemo^A4^* lane probably corresponds to a non-specific product caused by primer annealing to the transgenic vector sequence*.* The *rp49* control is also expressed at similar levels between these lines, but as expected, *hemotin* is not expressed in *CG7691GF;hemo^A4^* flies.

(D) Expression of *diptericin* mRNA in non-infected controls or after infection with *E.coli,* gram+ (*M. luteus)* or gram- (*E. clocae*) bacterial strains. After bacterial challenge, the expression of *diptericin* is up-regulated to a similar extent in both wild-type (Or-R), and *hemo^A4^* mutant flies. The expression of the *rp49* control remains constant in all different conditions.

(E) Pull down of HA-14-3-3ζ with Hemo- GFP in *Drosophila* pre-pupal hemocytes. Right panel: 14-3-3ζ interacts with hemo-GFP but not with GFP control. Left panel: Retention of Hemo-GFP and GFP control by the GFP beads.


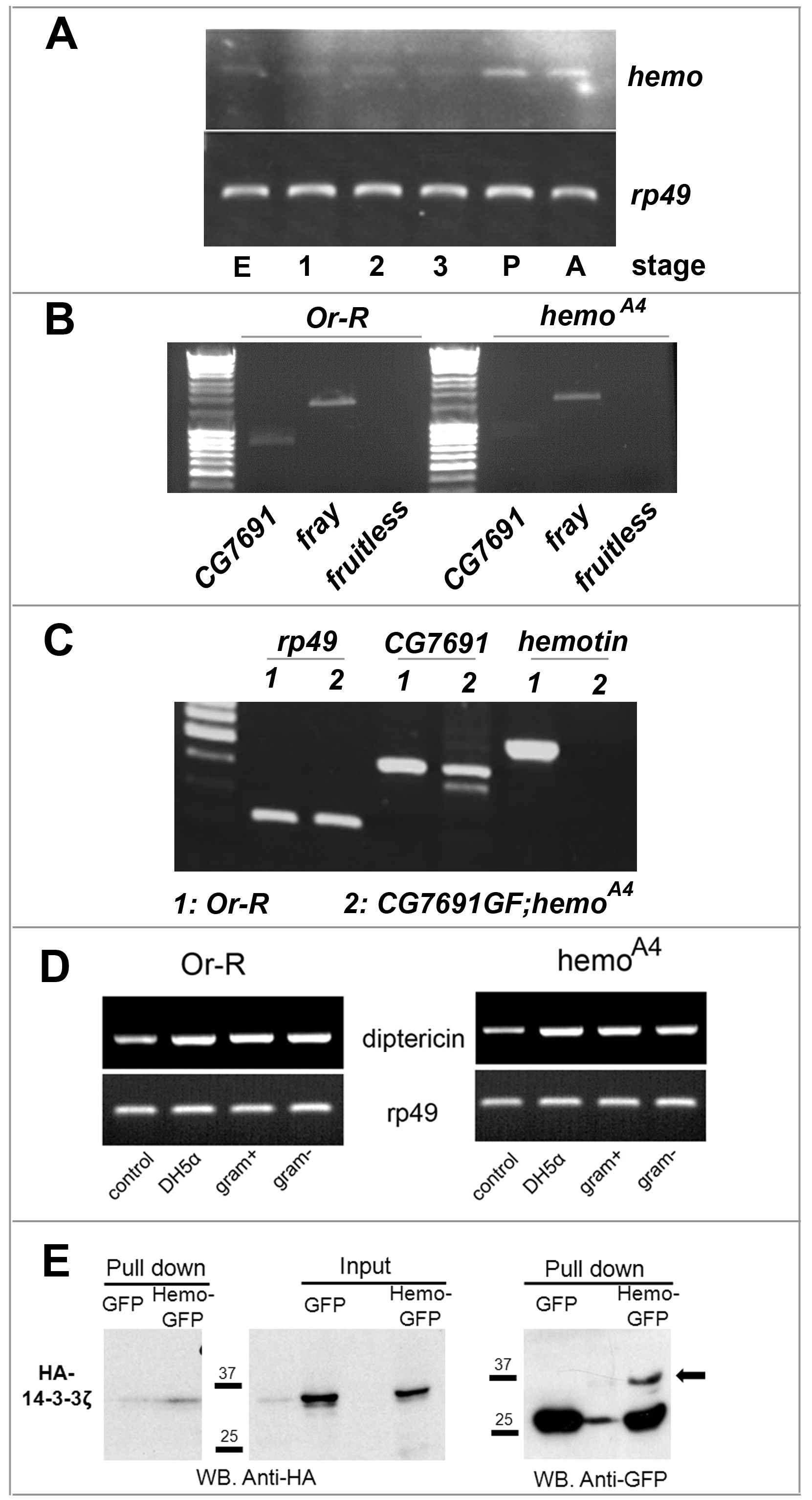

Supplement: S1 File — (DOCX) [file pbio.1002395.s008.docx]
