# Supplementary material for: Hemotin, a Regulator of Phagocytosis Encoded by a Small ORF and Conserved across Metazoans
Source: PLoS Biol. 2016 Mar 25;14(3):e1002395. doi: 10.1371/journal.pbio.1002395 (PMC4807881; doi:10.1371/journal.pbio.1002395)

**SUPPLEMENTARY FILE 2**

Hemo-GFP and ORF2-GFP expression in *Drosophila* S2 cells and phenotypic analysis of segmental nerves in *hemo^A4^* and *fray* allelic combinations.

(A-A’’) Expression of the carboxyl terminus tagged-ORF2 peptide from a full length *hemotin* transcript (ORF2-FL-GFP; green) and mCD8-RFP as a membrane marker (red) in transfected *Drosophila* S2 cells. (A) mCD8-RFP is detected in intracellular membranes (arrowhead). (A’) only very faint background GFP signal can be observed in a few cells (arrowhead). (A’) Merge image. Scale bar 20µm. Yellow dashed lines indicate cell outlines.

(B-B’’) Carboxyl terminus tagged-Hemo peptide (hemo-GFP) expression from a full length *hemotin* transcript and mCD8-RFP as a membrane marker in transfected *Drosophila* S2 cells labelled as in A. (B) mCD8-RFP labels cellular membranes (arrowhead). (B) hemo-GFP is expressed strongly and colocalizes with mCD8-RFP (arrowhead). B’’-Merge image. Scale bar 20µm. Yellow dashed lines indicate cell outlines.

(C) hemo-GFP peptide expression in *Drosophila* S2 cells is detected in intracellular vesicles (arrowhead).Scale bar 5µm.

(D) Expression of the carboxyl terminus GFP-tagged ORF2 peptide from a minigene containing only a short 5’UTR corresponding to the nucleotides between hemo-ORF and ORF2 in S2 cells. Strong ORF2-GFP expression is observed in intracellular compartments (arrowhead). Scale bar 5µm.

(E) Segmental nerves projecting from the ventral ganglion of a third instar *hemo^A4^* /*fray^PZ07551^* mutant larvae. These nerves have a smooth and compact shape (highlighted in orange). Scale bar 50µm.

(F) In third instar *fray* mutant larvae (*fray^PZ07551^/fray^R1^*), segmental nerves (highlighted in orange) have a bulged appearance (arrowhead), as described in Leiserson *et al.* (2000)[30], whereas *hemo^A4^*/ *fray^PZ07551^* larvae have wild type looking nerves, showing that the *hemo^A4^* deficiency complements this *fray* allele. Scale bar 50µm.


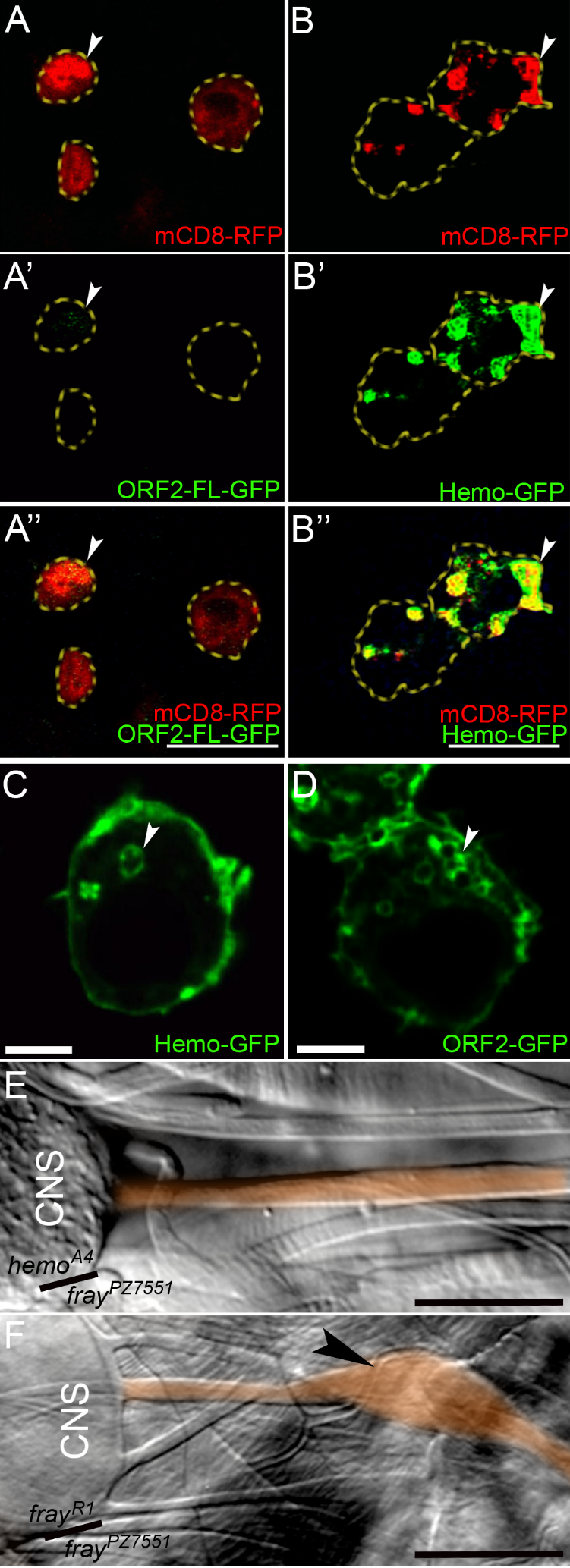

Supplement: S2 File — (DOCX) [file pbio.1002395.s009.docx]
