# Supplementary figures and images for: Hemotin, a Regulator of Phagocytosis Encoded by a Small ORF and Conserved across Metazoans
Source: PLoS Biol. 2016 Mar 25;14(3):e1002395. doi: 10.1371/journal.pbio.1002395 (PMC4807881; doi:10.1371/journal.pbio.1002395)

**SUPPLEMENTARY FILE 3**

**Percent Identity Matrix- created by Clustal2.1**


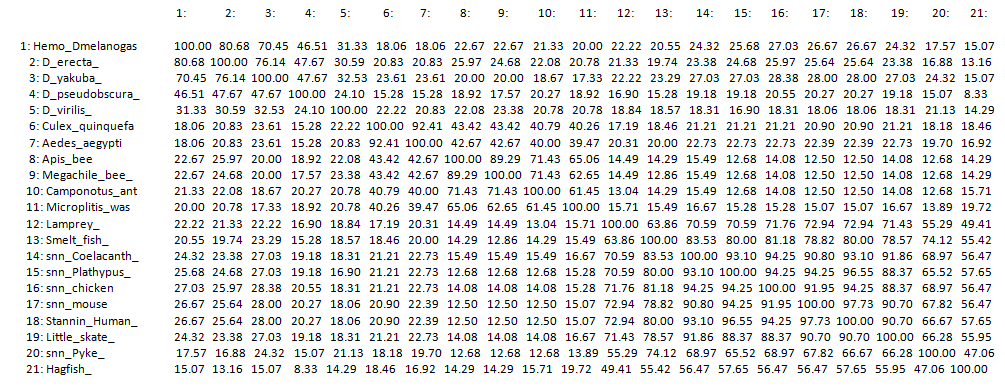

Supplement: S3 File — (DOCX) [file pbio.1002395.s010.docx]
